# Promoting plant-based eating in meat-centric meal contexts: a field study

**DOI:** 10.1017/S1368980023001763

**Published:** 2023-11

**Authors:** David Guedes, Vasco Brazão, Lisa Roque, Lúcia Campos, Cristina Godinho, Monica Truninger, Markus Vinnari, João Graça

**Affiliations:** 1 Iscte - Instituto Universitário de Lisboa, CIS_Iscte, Lisboa, Portugal; 2 Instituto de Ciências Sociais da Universidade de Lisboa (ICS-ULisboa), Lisboa, Portugal; 3 CLOO Behavioral Insights Unit, Porto, Portugal; 4 NOVA National School of Public Health, Public Health Research Center, Comprehensive Health Research Center, CHRC, NOVA University Lisbon, Lisbon, Portugal; 5 University of Helsinki, Helsinki, Finland; 6 University of Groningen, Groningen, The Netherlands

**Keywords:** Planetary health diet, Sustainability, Plant-based diets, Communal catering, Intervention

## Abstract

**Objective:**

Shifting from meat-centric to plant-rich diets may help to enable healthier and more sustainable food systems. Here we present the results of a 1-week intervention to promote plant-based eating in a meat-centric food context (i.e. canteen).

**Design:**

The intervention included environmental restructuring strategies (e.g. promotional materials and menu redevelopment) and improvements to the offer of plant-based meals. The evaluation (sales data; pre-registered) spanned 3 weeks prior to the intervention (baseline), 1 week during the intervention (immediate/short-term impact) and 3 weeks after the intervention (follow-up). Opinion surveys were also used to collect data with customers during the intervention.

**Setting:**

Canteen unit of a university campus in Portugal (Lisbon metropolitan area).

**Participants:**

In addition to sales data (baseline: 7965 meals; immediate/short-term: 2635 meals; follow-up: 7135 meals), we used opinion surveys to assess customers’ meal appraisals during the intervention (*n* = 370).

**Results:**

The odds of a sold meal being vegetarian were 24 % higher in the intervention week compared with the pre-intervention period [OR = 1·24, 95 % CI (1·10, 1·40)] and 9 % higher in the post-intervention period compared with the pre-intervention period [OR = 1·09, (95 % CI (1·00, 1·19)]. Survey data showed that vegetarian meals compared favourably to meat and fish alternatives in liking, sustainability and satiety.

**Conclusions:**

A short-term, theory-driven, operationally feasible intervention was effective in promoting increased plant-based meal choices in a collective meal context. Nevertheless, these changes were not entirely sustained over time. Future studies could test whether prolonged or more transformative interventions are necessary to unlock entrenched food practices more effectively in meat-centric collective meal contexts.

The global food system is a significant driver of environmental degradation. Feeding the world population is currently leading to substantial planetary damage, including climate change, depletion of water resources and disruption of aquatic and terrestrial ecosystems^(1)^. The food supply chain is one of the key generators of anthropogenic greenhouse gas emissions, with the largest slice of total agricultural emissions currently attributed to animal products^(1,2)^. Against this backdrop, urgent reforms of the current global food system have been called for, which necessarily include reducing food waste, improving production efficiency and promoting dietary changes^(2,3)^. With regard to dietary changes, in order to meet nutritional needs within planetary boundaries, reports from several organisations such as the United Nations Intergovernmental Panel for Climate Change and the EAT-Lancet commission have recommended shifts from meat-centric to increased plant-based food practices, especially in more economically developed countries^(2,4)^.

Notwithstanding the emerging scientific consensus around the expected benefits of increased plant-based eating, meat consumption levels have been rising steadily for the past five decades and are expected to increase by 14 % by 2030 according to recent projections^(5)^. This trend is levered not only by changes in the dietary patterns of developing countries but also by the reluctance of affluent societies to adhere to meat curtailment and increased plant-based eating^(6–9)^. A systematic review based on the COM-B^(10)^ system of behaviour identified several barriers in the capability, opportunity and motivation domains that still hinder dietary changes towards reduced meat consumption and more plant-based diets^(11)^. According to the review, barriers in the capability domain may include a lack of information and skills to prepare nutritionally balanced and appetising plant-based meals. Barriers in the opportunity domain may include hindrances in the social and physical or material context (e.g. antagonistic reactions and lack of social support from friends or family; limited access to plant-based meals in food-away-from-home settings). In turn, motivational barriers may include a lack of awareness and concern about the environmental impact of food production and consumption. These insights illustrate the multifaceted nature of food practices and suggest that knowledge and awareness of the benefits of dietary shifts may be insufficient to enable change. From that perspective, integrated, context-focused interventions that enable behaviour change within meal settings such as collective meal contexts (e.g. canteens, cafeterias) are needed to help promote sustainable food transitions^(11–13)^.

## Collective meal contexts as drivers of dietary change

Myriad personal and contextual factors influence food choices. While individual-centered approaches may focus mainly on reflective motivations and deliberate thinking processes, such as values or preferences, food choices are also influenced by situational and contextual determinants, such as the social or physical environment^(14)^. In recent years, there has been an increased interest in how situational and contextual factors may shape food choices, along with developments in the nudging and food choice architecture literature^(15–17)^. Nudging refers to initiatives that aim at influencing behaviour in a predictable way without imposing or restricting individual choice^(15,17)^. These initiatives aim at subtly influencing decision making, for instance, by changing the position of food products (e.g. placing the product to be incentivised closer to the consumer), changing default settings (e.g. making a healthy side as the default garnish for a meal) or adding verbal or visual cues to shift attention towards a desired food item (e.g. adding attention-grabbing stickers next to the more sustainable products). Reflective motivations, such as health concerns or orientations towards sustainable consumption, are often at odds with more automatic determinants such as habits and cravings. In today’s food systems and food environments, it is common for unhealthy and less sustainable meal choices to be the more convenient or cheaper options. To address this paradox, there have been efforts to reconcile automatic and deliberate motivations by implementing context-based initiatives that may offer simple, unobtrusive and cost-efficient means for promoting healthier and more sustainable eating^(17–22)^.

## The current study: aims and hypotheses

Collective meal contexts, such as canteens, restaurants or cafeterias, may play a role in sustainable food transitions. Given their potential for unlocking large-scale change, these settings offer promising opportunities to tackle meat-centric practices, such as the provision of a standard offer of dishes where meat and fish are the centre of the plate protein sources and there is limited choice or availability of appropriate plant-based meal alternatives^(12,23,24)^. Here we aimed to test the potential of a one-week, theory-driven, operationally feasible intervention in a canteen setting to promote vegetarian meal choices (including ovo-lacto-vegetarian and vegan meals). We hypothesised that 1) the intervention would increase the proportion of vegetarian meals purchased during the 1-week intervention compared with the 3 weeks prior to intervention and 2) the increase in vegetarian meal choice would remain above baseline levels 3 weeks after intervention.

## Methods

This study was pre-registered on AsPredicted.org: https://aspredicted.org/jp34m.pdf. All materials (original and translated) as well as analysis code and reports are available on OSF: https://osf.io/nuhkv/.

### Intervention, materials and procedure

The intervention took place in a canteen unit of a university campus in Portugal (Lisbon metropolitan area; approximately 8000 students). The canteen’s customer base was students, and the menu offered a choice between three meal options per day – one with meat, one with fish and one vegetarian, which changed daily. The evaluation began 3 weeks prior to the implementation of the intervention by collecting baseline sales data, continued during the implementation and finished 3·5 weeks after the implementation. The implementation period spanned 1 working week, framed as a ‘sustainability week’ initiative promoted by the university at the canteen.

According to the COM-B system of behaviour^(10)^ applied to meat reduction and plant-based eating^(11)^, dietary change may be framed as resulting from the interplay between capability, opportunity and motivation variables. Multicomponent interventions in collective meal settings allow for activating variables at all three domains of the COM-B system, thus maximising intervention potential. Hence, our intervention activities included practical training for the kitchen staff and a promotional campaign that included modifications to the physical and social environment. Besides sales data, a brief opinion survey was distributed in the canteen during the intervention period. All promotional materials were withdrawn from the canteen after the intervention period and follow-up data collection (sales data) took place for 3·5 weeks post-intervention. The intervention activities were planned based on previous research relevant to meat reduction and collective meal contexts^(12,16,18,21,25–28)^ and a review of capability, opportunity and motivation variables to promote increased plant-based eating^(11)^. An overview of the intervention activities is presented below, and a summary with links to the COM-B system is presented in Table 1.

**Table 1:** Overview of intervention activities and their relation to the COM-B system

Intervention strategy	Intervention activity	Capability (Staff)	Opportunity (Consumers)	Motivation (Consumers)
Training: canteen staff	Practical workshop with the staff about plant-based cooking	x	x	
Visual communication materials: posters	Giving visibility to the ‘Sustainability week’		x	x
	Encouraging users to try out new (improved) plant-based meals		x	x
	Giving visibility to the environmental impact of food			x
Menu redevelopment	Reordering meals to highlight the vegetarian option		x	
	Using sensory and hedonic language cues to display the vegetarian option			x

#### Training

A 2-day workshop was held with the canteen kitchen staff before the intervention period. This activity aimed to provide the staff with relevant knowledge and practical skills for improving the sensory attributes and nutritional profile of plant-based meals served in the canteen. The workshop was conducted by a chef with extensive experience in plant-based cooking and training staff in school and university settings. The workshop included an overview of the relevance of promoting increased plant-based eating in canteens (i.e. sustainability transitions at the university level), basic principles of plant-based nutritional literacy (e.g. nutritionally balanced plant-based meals) and practical demonstrations of how to improve the visual and sensory appeal of plant-based meals (e.g. combining spices and herbs to improve tastefulness), with a learning-by-doing approach *in situ* (kitchen) focusing on meals suitable to the university canteen setting (e.g. curry, bean stew, stroganoff). To uphold feasibility, the workshop focused mostly on improving the current offer instead of aiming for structural changes in the meals portfolio of the canteen. Staff members reported to our team that they felt capable of using their new knowledge and skills to improve the current offer, but the assessment did not track changes in the palatability and nutrient content of the meals before, during and after the intervention. The scientific accuracy and validity of the workshop materials and activities were also not subjected to external assessment.

#### Communication materials

Three types of visual communication materials were developed to restructure the physical environment. A total of eighteen posters were placed in visible locations inside the canteen and nearby access sites. The first set of posters gave visibility to the ‘Sustainability Week’ initiative. This communication product was designed to call attention to the initiative and express a normative/institutional endorsement of sustainable food practices. A second set of posters informed canteen users that an intervention was in place to improve the quality of vegetarian meals and encouraged them to try out the meals and give their opinion (‘Did you know that we’re improving our vegetarian meals? Try them and tell us what you think’). Finally, the third set of posters sought to raise awareness about the environmental footprint of food and different food products. The posters included a QR code redirecting users to a footprint calculator which allowed them to compare the sustainability metrics of different food products.

#### Menu redevelopment

The canteen announced the daily menus via the university website, and no longer used a physical menu for this purpose. We redeveloped the online menu and developed additional physical menus such that the vegetarian option was presented above the meat and fish options. These physical menus were placed so users in line for food would see them immediately before seeing the food on offer and having their first interaction with the server. The vegetarian option was further emphasised in the physical menu by increasing the font size and placing a leaf-like visual icon alongside the meal name was presented throughout the intervention week as the ‘suggestion of the day’. Vegetarian options were also renamed to improve the sensory and hedonic appeal. For example, ‘vegetable curry with rice’ was renamed ‘creamy and aromatic vegetable curry with rice’ (sensory labelling). The new meal names were developed with the kitchen staff to promote the staff’s involvement and guarantee that the sensory descriptions were aligned with the actual sensory attributes of the meals. When no improvement was possible, or the dish was already well known and in high demand, the corresponding name was kept the same.

### Measures

#### Meal choice

Data on sales of vegetarian, fish and meat options were collected daily for each meal (lunch, dinner and ‘snack’^1^) from 1 September to 22 December 2021. As specified in the pre-registration, we used data from November and December to evaluate the intervention: the baseline (pre-intervention) period lasted from 1 November to 20 November (3 weeks), the intervention period lasted from 22 November to 26 November (1 week), and the follow-up period lasted from 29 November until the last day before winter holidays, 22 December (3·5 weeks). Data were not collected on weekends or national holidays, as the canteen was closed. Other than Saturdays and Sundays, this included the following holidays: 1 November, 1 December and 8 December.

#### Opinion survey

A brief survey was made available throughout the intervention week. The survey was programmed in Qualtrics and distributed via QR codes placed in visible places on the canteen tables. Additionally, one member of the research team was present in the canteen during lunchtime encouraging users to fill out the online questionnaire. The survey included basic socio-demographic questions (age and gender), one meal choice item (meat, fish, vegetarian and other) and one awareness check item (‘Did you notice that a Sustainability Week initiative was taking place at the canteen?’) with three response options (No, Yes and Only today). Participants evaluated each meal on five dimensions of liking (1 = *I didn’t like it very much* to 5 = *I liked it very much*), healthfulness (1 = *Not very healthy* to 5 = *Very healthy*), naturalness (1 = *Not very natural* to 5 = *Very natural*), sustainability (1 = *Not very sustainable* to 5 = *Very sustainable*) and satiety (1 = *Not very satiating* to 5 = *Very satiating*).

### Sales data and participants

Sales data were obtained for 7965 meals during the baseline period, 2635 during the intervention stage and 7135 during follow-up. We also received 370 responses to the online survey during the intervention week. Since the same participant could complete the survey more than once on different intervention days, basic socio-demographic statistics are presented separately for each intervention day (Table 2). The average age of respondents was 22·9 years (sd = 8·6 years), 168 respondents identified as men, 131 as women, ten chose the option ‘other’, nine chose the option ‘prefer not to say’ and fifty-two responses were missing.

**Table 2:** Basic socio-demographic characteristics per intervention day

	Monday (*n* 93)	Tuesday (*n* 63)	Wednesday (*n* 44)	Thursday (*n* 90)	Friday (*n* 80)	Total (*n* 370)
Socio-demographic data *n*	80	58	34	74	66	312
Age						
Mean	24·1	23·1	25·3	21·0	22·2	22·9
sd	10·0	7·7	10·0	6·4	8·1	8·6
Female						
*n*	24	23	18	36	30	131
%	30·0 %	39·7 %	50·0 %	47·4 %	44·1 %	42·0 %

### Data analyses

All data analyses were conducted in R, using RStudio^(29)^ as an interface for literate programming inside RMarkdown documents. The data wrangling and analysis code as well as the corresponding reports are available at https://osf.io/nuhkv/ ^2^. For the survey data, descriptive, exploratory analyses were conducted, to compare students’ perceptions of the different meals with each other. The sales data were analysed according to the pre-registration. Our main analysis compared the proportion of vegetarian meals sold (1) in the 3 weeks before the intervention, (2) in the week of the intervention and (3) in the 3·5 weeks after the intervention using a simple logistic regression with the number of vegetarian meals sold at lunch (weighted by the total number of meals at lunch) being predicted by condition. The code for the model specification can be found at https://osf.io/snjvr/ on page 7. The estimated model can be equivalently described with the following equations:

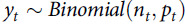




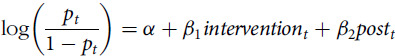




where *y*_*t*_ is the number of vegetarian lunch meals sold on day *t*, *n*_*t*_ is the total number of lunch meals sold on day *t*, *p*_*t*_ is the probability that a given sold meal is vegetarian, *α* is an intercept, corresponding to the log odds of a meal being vegetarian during the pre-intervention period, *β*_*1*_ represents the change in log odds for the intervention period, *β*_*2*_ represents the change in log odds for the post intervention period, *t* corresponds to the day of measurement and *intervention*_*t*_ and *post*_*t*_ are indicator variables equal to 1 when a given day belongs to the respective period and 0 otherwise.

To check the robustness of our findings, the main model was fit three more times to allow for sensitivity analyses excluding: (1) the first day of the intervention week (Monday, 22 November) because we reprinted the posters to make them more visible (i.e. same content but larger dimension) from the second day onwards (Tuesday, 23 November); (2) the last 3 days of the post-intervention assessment period (Monday to Wednesday, 20–22 December) as they formed an incomplete week right before the Christmas holidays and (3) all days mentioned in (1) and (2).

To take advantage of the full dataset (which included the two meal types beyond our main focus), we further specified two multilevel models (the first with random intercepts by meal, the second with random intercepts by meal and random slopes for condition by meal) and fit them to the complete intervention data, without omitting any meal type. As the second model showed singular fit, we ran the three sensitivity analyses mentioned in the previous paragraph with the first multilevel model only. For parsimony, these results are not presented in the main text, but can be consulted in our supplementary materials.

## Results

### Sales data: intervention effectiveness

In total, 17 735 meal sales were recorded during November and December, of which 13 072 were lunches. As per the pre-registration our primary model used lunch data. Table 3 shows the breakdown of these sales by meal type and intervention period.

**Table 3:** Number and proportion of lunch meals sold by type of meal and time period

	Meal type					
	Vegetarian		Meat		Fish	
Period	*n*	%	*n*	%	*n*	%
Pre-intervention, 3 weeks	1253	21·3	3326	56·5	1303	22·2
Intervention, 1 week	461	25·1	950	51·7	425	23·1
Post-intervention, 3·5 weeks	1121	22·8	2579	48·2	1554	29·0

#### Planned primary analyses

Logistic regression was used to analyse the relationship between the time period (entered as a factor variable with the pre-intervention period as the reference category) and the proportion of vegetarian meals sold at lunch (weighted by the total number of meals sold within that time period). Regression results are presented in Table 4. We found that the odds of a sold meal vegetarian were 24 % higher in the intervention week compared with the pre-intervention period (OR = 1·24, 95 % CI (1·10, 1·40)) and 9 % higher in the post-intervention period compared with the pre-intervention period (OR = 1·09, (95 % CI (1·00, 1·19)). The probability of a sold meal being vegetarian within each time period was estimated to be: 21·3 % (95 % CI (20·3 %, 22·4 %)) pre-intervention, 25·1 % (95 % CI (23·2 %, 27·1 %)) during the intervention and 22·8 % (95 % CI (21·7 %, 23·9 %)) post-intervention.

**Table 4:** Primary logistic regression results

Effect	OR	se	95 % CI	*P*-value
Intercept	0·27	0·032	0·25, 0·29	< 0·001
Intervention	1·24	0·063	1·10, 1·40	< 0·001
Post-intervention	1·09	0·046	1·00, 1·19	0·055

OR = Odds Ratio, se = Standard Error, CI = Confidence Interval.

#### Planned sensitivity analyses

We ran the same model above on data that excluded either (1) just the first day of the intervention week, (2) just the last 3 days of the post-intervention period or (3) both the first day of the intervention week and the last 3 days of the post-intervention period. Table 5 summarises the results, showing the estimated OR and associated *P* value for the coefficient comparing the intervention period with the pre-intervention period for each sensitivity analysis. In summary, our main results were sensitive to the exclusion of the first day of the intervention week, but not the exclusion of the last 3 days of the post-intervention period. The remaining results for these models, including the multilevel models mentioned in Section 2·4, are available at https://osf.io/snjvr/.

**Table 5:** Comparison between different sensitivity analysis specifications on the main effect of interest

Specification	Model	OR	95 % CI	*P* value
1	Single level	1·14	1·00, 1·31	0·050
2	Single level	1·24	1·10, 1·40	< 0·001
3	Single level	1·14	1·00, 1·31	0·050

#### Survey data: awareness about the intervention and appraisal of vegetarian meals

Survey response referred to a total of 188 meat meals, forty-nine fish meals and 113 vegetarian meals; five meals were reported as ‘other’, and fifteen meal responses were missing. The proportion of respondents who noticed the intervention grew steadily throughout the week; however, on the last day of the intervention, 22·5 % of respondents claimed not to have noticed the intervention yet, while a further 15 % left the question unanswered (Table 6 shows the complete results for this question).

**Table 6:** Number and proportion of respondents who had noticed the intervention throughout the week

	No, had not noticed		Yes, had already noticed		Yes, noticed today		NA (Missing responses)	
	*n*	%	*n*	%	*n*	%	*n*	%
Monday	43	46·2	4	4·3	34	36·6	12	12·9
Tuesday	24	38·1	12	19·1	23	36·5	4	6·4
Wednesday	14	31·8	8	18·2	15	34·1	7	15·9
Thursday	28	31·1	21	23·3	28	31·1	13	14·4
Friday	18	22·5	33	41·3	17	21·3	12	15·0

In general, those who had consumed vegetarian meals liked their meals more and considered them more sustainable and filling than those who had consumed meat or fish, while fish meals were rated as healthier and more natural than the other types of meal. For example, 34·5 % of those who consumed a vegetarian meal reported having liked it ‘a lot’, while the percentage was 10·1 % for meat meals and 12·2 % for fish meals. Table 7 displays the means and standard deviations for each item and meal type. Unplanned inferential analyses with further comparisons of meal ratings are provided in the online supplementary materials (https://osf.io/q7vyb/).

**Table 7:** Respondents’ ratings of each meal

	Liking		Sustainable		Healthy		Natural		Satiating	
	Mean	sd	Mean	sd	Mean	sd	Mean	sd	Mean	sd
Vegetarian	4·14	0·89	3·72	1·06	3·69	1·03	3·38	1·01	3·96	1·14
Fish	3·60	0·79	3·20	0·88	3·83	1·03	3·51	1·10	3·52	1·43
Meat	3·51	0·81	3·00	0·98	3·20	0·83	3·08	1·01	3·62	1·06

## Discussion

Dietary changes are critical to ensuring healthier and more sustainable food systems^(4)^. Interventions to promote increased plant-based eating in collective meal contexts may help scale and accelerate these dietary changes^(12,22,30–38)^. The present work reported the results of a brief multicomponent intervention for increasing plant-based eating in a university canteen. The intervention consisting of training, communication materials, and menu redevelopment led to a significant increase in the sales of vegetarian meals during the intervention week, but this difference was not fully sustained in the post-intervention period.

Overall, these results align with previous evidence suggesting that multi-component interventions, activating different behaviour change levels, may promote (at least immediate/short term) dietary shifts^(39–41)^. This is also consistent with the notion that transitioning to more plant-based eating can be facilitated by integrated approaches addressing variables in the capability, opportunity and motivation domains^(11,12)^. The intervention reported here consisted of a short-term, theory-driven, operationally feasible set of activities that may be transferable to other collective meal contexts. Indeed, feasibility and cost-effectiveness have been arguments in favour of interventions based on choice architecture principles – but see evidence reporting null results, too^(42)^. Understanding what works in multicomponent interventions is often challenging as it obviates the estimation of the effectiveness of individual intervention components. It is also important to ensure that intervention activities are coherent and complementary to avoid mismatches potentially compromising effectiveness. For instance, using sensorial simulation language on the menu may influence individuals’ expectations towards sensory properties of meals, but this strategy may backfire if those expectations are not met (or are rather disconfirmed)^(43)^.

One key feature of the current intervention was to implement a training workshop with canteen staff to improve the sensory attributes of plant-based meals offered in the canteen. Indeed, it is reasonable to expect that the capacity of catering staff and service managers to provide appetising and nutritionally balanced plant-based meals may indirectly influence consumers’ motivation and opportunity to choose plant-based options in these meal settings. Besides targeting capability variables to improve service provision, the current intervention also aimed to encourage consumers to choose plant-based meal options by making these options more visible and raising awareness about the environmental impact of food. Taken as a whole, these activities effectively increased the proportion of vegetarian meals sold during the intervention period. Nevertheless, this increase was relatively modest, not fully sustained in the post-intervention period and not sufficient to challenge the dominance of meat in the canteen’s food practices, as meals with meat continued to be the most favoured choice in this setting even during the intervention period.

One possible interpretation of these findings is that the intervention would require a more extended implementation period to yield more substantial changes in this setting – which would also enable attentive tinkering and careful adjustments of the activities throughout time to strengthen its potential impact^(20)^. The motivation for this study was to examine the potential of a short-term, theory-based, operationally simple intervention to promote changes in a meat-centric meal context. Yet, it is likely that the limited duration of these activities was insufficient to foster more pronounced impacts, for instance, in users’ familiarity with the plant-based options and positive representations within the academic community through word-of-mouth. As such, the findings presented here should be interpreted in light of the intervention dosage and not only the intervention components *per se*.

Another interpretation of the current findings is that our integrated approach to enable dietary change in this meal context was promising but too incremental to unlock consumers’ entrenched meat-centric habits and expectations more effectively. On the one hand, it is possible that more transformative approaches may require greater involvement of provision services and be prone to generating more operational hindrances. On the other hand, overcoming key barriers and enabling plant-based eating may require more structural food service transformations, such as increasing and diversifying the range of plant-based options, reformulating pricing schemes, or altering the availability of meat-centric options. Indeed, recent reviews suggest that mobilizing multiple intervention levels and components in collective meal settings contributes to more consistent outcomes^(12,41)^.

Our interpretations remain open, as the intervention had a short-term nature, did not include a process evaluation to systematically collect qualitative inputs from the actors involved in or affected by the intervention (e.g. service manager, staff and consumers) and did not track changes in the palatability and nutrient content of the meals. Furthermore, the visual communication materials were removed after the intervention period, whereas the gains resulting from staff training were likely sustained to an undetermined extent beyond the intervention. The repeated-measures design of the study also requires us to hedge our conclusions, given that without a comparison canteen we cannot rule out that changes in consumption were due to other transient determinants, rather than the intervention components themselves. Another limitation of the current study is that although our reliance on sales data provided a relatively objective measure of food choices, it could not inform us about consumers’ perceptions of meal alternatives. Consumers who chose vegetarian meals during the intervention week reported liking their meals more and perceiving them as healthier and more sustainable than those who opted for meat/fish options. However, since no baseline data were available for these measures, it is unclear whether these results may be attributable to the intervention. It is also noteworthy that participants’ awareness of the intervention initiatives increased throughout the intervention period, yet almost a quarter of respondents had not noticed that the intervention was taking place after five days of implementation. Some activities could be expected to remain outside the scope of conscious awareness (e.g. menu redevelopment). However, the intervention components that targeted more deliberate motivation processes (e.g. awareness and concern about the environmental impact of food) would likely require a more active engagement from participants to be effective. Future intervention studies could seek to ensure that the materials and activities targeting conscious awareness and reflective motivation are noticeable even to more absent-minded consumers. Moreover, combining theoretical frameworks may be a promising avenue for developing more transformative interventions, in which behavioural approaches are complemented with social practice theories^(44,45)^ to better account for the complexities of how people interact with the wider social and cultural context^(46)^.

## Conclusion

The results presented here suggest that a short-term, operationally feasible intervention based on choice architecture and staff training was effective in promoting increased plant-based meal choices in a collective meal context. However, these changes did not challenge meat’s dominance in this meal setting. Future studies could test whether the impact of integrated approaches such as the one implemented here could be expected to increase with more extended implementation periods, or whether more transformative interventions would be necessary to unlock entrenched food practices more effectively in meat-centric collective meal contexts.

## Footnotes

1
The canteen offered two lines with the same meals during lunch time, one for students and one for staff members. The one labelled ‘lunch’ was used by students and is the focus of our intervention, while the one labelled ‘snack’ was used by staff.

2
The reports are not fully reproducible because we do not have permission to store the data on an open repository. However, we provided a .zip folder containing all the files we are able to share and the original folder structure for the R Project. To test the code for the sales analysis, we provide a synthetic dataset that contains the same variables as our original data, and an RMarkdown document called ‘Sales Analysis Synthetic Data’. Results from the synthetic data report will not match our reported results, as the data serve only to show that the code runs.

## Supplementary material

For supplementary material accompanying this paper visit https://doi.org/10.1017/S1368980023001763. click here to view supplementary material
