# Supplementary material for: Promoting plant-based eating in meat-centric meal contexts: a field study
Source: Public Health Nutr. 2023 Sep 15;26(11):2619–27. doi: 10.1017/S1368980023001763 (PMC10641616; doi:10.1017/S1368980023001763)
Supplement: Supplementary file 1 [file S1368980023001763sup.zip › S1368980023001763sup001.pdf]

Questionnaire Report

SUSTAINMEALS

Document last updated: 2022-10-28 16:29:56

```
library(here)
library(tidyverse)
library(readxl)
library(gt)
library(gtsummary)
library(ggpubr)
library(knitr)
library(sessioninfo)

options(digits = 4)
```

```
responses <- readRDS(here::here("01_wrangling", "data_questionnaire.RDS"))
```

```
total_n <- nrow(responses)

tbl_resp_by_date_raw <- responses %>%
  dplyr::filter(
    StartDate_day > "2021-11-21"
  ) %>%
  dplyr::count(Day)

tbl_resp_by_date_meal_raw <- responses %>%
  dplyr::filter(
    StartDate_day > "2021-11-21"
  ) %>%
  dplyr::count(
    Day,
    Meal
  )
```

This report summarizes the results of the questionnaire handed out as part of the intervention.

There were 370 valid responses to the questionnaire during the week of 22 to 26th of November 2021.

Table 1: Number of responses by day and meal

```
#set tables to English
theme_gtsummary_language(
  language = "en",
  decimal.mark = ".",
```

```
  big.mark = NULL,
  iqr.sep = NULL,
  ci.sep = NULL,
  set_theme = TRUE
)

tbl_resp_by_date_meal_raw %>%
  tidyr::pivot_wider(
    names_from = Meal,
    values_from = n
  ) %>%
  gt::gt() %>%
   tab_spanner(
    label = "Meal",
    columns = c(lunch, dinner, "NA")
  )
```

	Meal		
Day	lunch	dinner	NA
Monday	75	11	7
Tuesday	62	1	NA
Wednesday	43	1	NA
Thursday	85	2	3
Friday	72	3	5

Most responses occured during lunch.

Table 2: Age distribution by day

```
#age
sumstats_age_data <- responses %>%
  select(Age, Day) %>%
  gtsummary::tbl_summary(by = Day,
    type = all_continuous() ~ "continuous2",
    statistic = all_continuous() ~ c("{N_nonmiss}",
                                     "{mean} ({sd})",
                                     "{median} ({p25}, {p75})",
                                     "{min}, {max}"),
    digits = all_continuous() ~ 2,
    missing = "no"
  ) %>%
  italicize_levels()%>%
  modify_spanning_header(
    list(
      all_stat_cols() ~ "**Date**"
    )
  )

sumstats_age_data
```

**Characteristic**	**Monday**, N = 93	**Tuesday**, N = 63	**Wednesday**, N = 44	**Thursday**, N = 90	**Friday**, N = 80
Age					
N	80.00	58.00	34.00	74.00	66.00
Mean (SD)	24.09 (10.02)	23.07 (7.70)	25.32 (10.01)	21.04 (6.84)	22.15 (8.08)
Median (IQR)	21.00 (18.00, 24.00)	21.00 (19.25, 23.00)	21.00 (19.00, 27.75)	19.00 (18.00, 21.00)	20.00 (18.00, 22.75)
Range	17.00, 58.00	18.00, 58.00	18.00, 61.00	17.00, 62.00	12.00, 70.00

Table 3: Age distribution by meal

```
sumstats_age_prato <- responses %>%
  select(Age, Meal) %>%
  tbl_summary(by = Meal,
    type = all_continuous() ~ "continuous2",
    statistic = all_continuous() ~ c("{N_nonmiss}",
                                     "{mean} ({sd})",
                                     "{median} ({p25}, {p75})",
                                     "{min}, {max}"),
    digits = all_continuous() ~ 2,
    missing = "no"
  ) %>%
  italicize_levels()%>%
  modify_spanning_header(
    list(
      all_stat_cols() ~ "**Meal**"
    )
  )
sumstats_age_prato
```

**Characteristic**	**lunch**, N = 337	**dinner**, N = 18
Age		
N	299.00	13.00
Mean (SD)	22.90 (8.72)	22.85 (4.36)
Median (IQR)	20.00 (18.00, 23.00)	22.00 (20.00, 26.00)
Range	12.00, 70.00	18.00, 33.00

Table 4: Gender distribution by date

```
#gender
sumstats_gender_data <- responses %>%
  select(Gender, Day) %>%
  tbl_summary(by = Day,
              digits = everything() ~ 2)%>%
  italicize_levels()%>%
  modify_spanning_header(
    list(
      all_stat_cols() ~ "**Date**"
```

```
    )
  )

sumstats_gender_data
```

**Characteristic**	**Monday**, N = 93	**Tuesday**, N = 63	**Wednesday**, N = 44	**Thursday**, N = 90	**Friday**, N = 80
Gender					
female	24.00 (30.00%)	23.00 (39.66%)	18.00 (50.00%)	36.00 (47.37%)	30.00 (44.12%)
male	50.00 (62.50%)	32.00 (55.17%)	17.00 (47.22%)	38.00 (50.00%)	31.00 (45.59%)
other	4.00 (5.00%)	1.00 (1.72%)	0.00 (0.00%)	1.00 (1.32%)	4.00 (5.88%)
prefer not to say	2.00 (2.50%)	2.00 (3.45%)	1.00 (2.78%)	1.00 (1.32%)	3.00 (4.41%)
Unknown	13	5	8	14	12

Descriptives

```
## in-text objects for sample description, reduced no. of decimal places for mean and sd of age to 1.
#mean sd of age

mean_age1 <- mean(responses$Age, na.rm = TRUE)

mean_age <- format(round(mean_age1, 1), nsmall = 1)

sd_age1 <- sd(responses$Age, na.rm = TRUE)

sd_age <- format(round(sd_age1, 1), nsmall = 1)
```

Mean age was 22.9 years, with a standard deviation of 8.6 years

We had the following gender distribution:

```
responses %>% dplyr::count(Gender) %>% knitr::kable()
```

Gender	n
female	131
male	168
other	10
prefer not to say	9
NA	52

Participants consumed the following meals:

```
responses %>% dplyr::count(Dish) %>% knitr::kable()
```

Dish	n
other	5
meat	188
fish	49
vegetarian	113
NA	15

Visualization

The following graph displays responses to the question "Did you notice that"Sustainability Week" is happening in the University Canteen?"

```
plot_1 <- responses %>%
  ggplot(aes(x = Day, fill = Noticed)) +
  geom_bar(position = "fill") +
  scale_fill_manual(values = c("#fe6f61", "#26315d", "#a6cfe5")) +
  scale_x_discrete("Day", labels = c("Monday" = "Mon","Tuesday" = "Tue", "Wednesday" = "Wed","Thursday"
theme_minimal() +
    theme(axis.title.y = element_text(angle = 0, vjust = 0.5, hjust = 1)) +
  labs(
    x = "Day",
    y = "Responses"
  )

plot_1
```

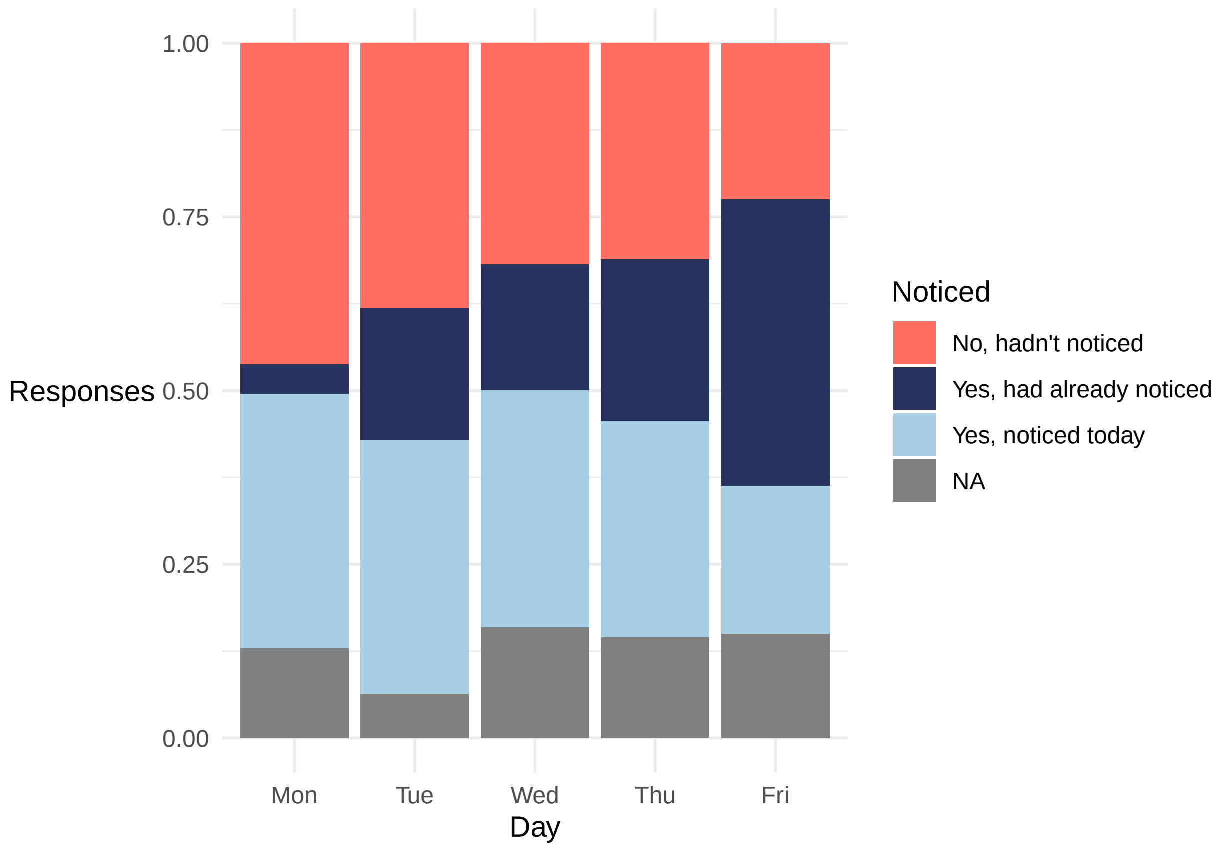

Proportions within each day:

```
responses %>%
  count(Day, Noticed) %>%
  group_by(Day) %>%
  mutate(prop = n/sum(n)) %>% knitr::kable()
```

Day	Noticed	n	prop
Monday	No, hadn't noticed	43	0.4624
Monday	Yes, had already noticed	4	0.0430
Monday	Yes, noticed today	34	0.3656
Monday	NA	12	0.1290
Tuesday	No, hadn't noticed	24	0.3810
Tuesday	Yes, had already noticed	12	0.1905
Tuesday	Yes, noticed today	23	0.3651
Tuesday	NA	4	0.0635
Wednesday	No, hadn't noticed	14	0.3182
Wednesday	Yes, had already noticed	8	0.1818
Wednesday	Yes, noticed today	15	0.3409
Wednesday	NA	7	0.1591
Thursday	No, hadn't noticed	28	0.3111
Thursday	Yes, had already noticed	21	0.2333
Thursday	Yes, noticed today	28	0.3111
Thursday	NA	13	0.1444
Friday	No, hadn't noticed	18	0.2250

Day	Noticed	n	prop
Friday	Yes, had already noticed	33	0.4125
Friday	Yes, noticed today	17	0.2125
Friday	NA	12	0.1500

The following graph shows the proportions of meal types consumed per day.

```
plot_2 <- responses %>%
  ggplot(aes(x = Day, fill = Dish)) +
  geom_bar(position = "fill") +
  scale_fill_manual(values = c("#f4e79e", "#26315d", "#a6cfe5", "#fe6f61")) +
  scale_x_discrete("Dia", labels = c("Monday" = "Mon","Tuesday" = "Tue", "Wednesday" = "Wed","Thursday"
  theme_minimal() +
  theme(axis.title.y = element_text(angle = 0, vjust = 0.5, hjust = 1)) +
  labs(,
    x = "Dish",
    y = "Responses"
  )

plot_2
```

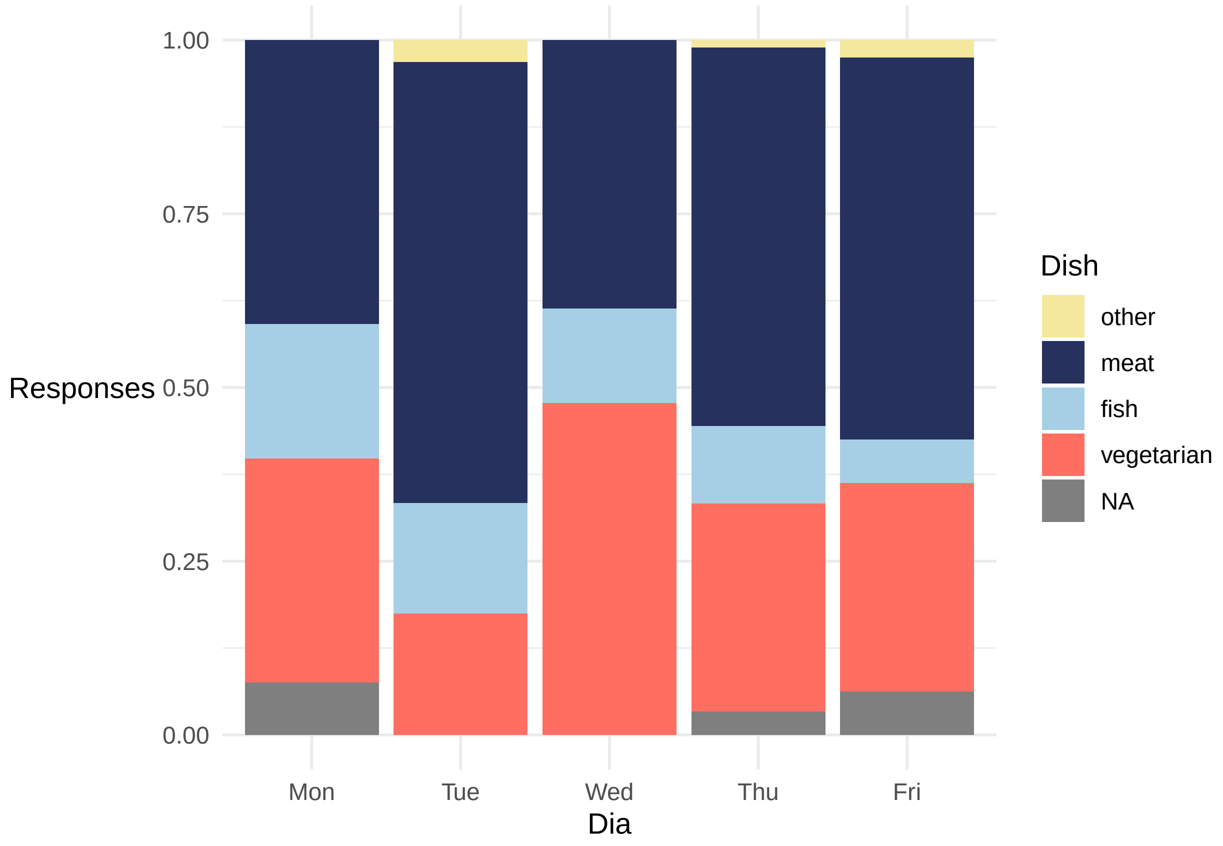

Proportions within each day:

```
responses %>%
  count(Day, Dish) %>%
  group_by(Day) %>%
  mutate(prop = n/sum(n)) %>% knitr::kable()
```

Day	Dish	n	prop
Monday	meat	38	0.4086
Monday	fish	18	0.1935
Monday	vegetarian	30	0.3226
Monday	NA	7	0.0753
Tuesday	other	2	0.0317
Tuesday	meat	40	0.6349
Tuesday	fish	10	0.1587
Tuesday	vegetarian	11	0.1746
Wednesday	meat	17	0.3864
Wednesday	fish	6	0.1364
Wednesday	vegetarian	21	0.4773
Thursday	other	1	0.0111
Thursday	meat	49	0.5444
Thursday	fish	10	0.1111
Thursday	vegetarian	27	0.3000
Thursday	NA	3	0.0333
Friday	other	2	0.0250
Friday	meat	44	0.5500
Friday	fish	5	0.0625
Friday	vegetarian	24	0.3000
Friday	NA	5	0.0625

The following graphs show participants' ratings of each meal type. After each graph there is a table with the corresponding values.

```
# Liking - recode likert data
responses <- responses %>% mutate(Like = recode(Like,
  "1" = "Liked little",
  "2" = "2",
  "3" = "3",
  "4" = "4",
  "5" = "Liked a lot"
))

# reorder factor levels

responses$Like <- factor(responses$Like, levels = c("Liked a lot","4", "3", "2", "Liked a little"), ord

# Healthy

responses <- responses %>% mutate(Healthy = recode(Healthy,
  `1` = "Not very healthy",
  `2` = "2",
  `3` = "3",
  `4` = "4",
```

```
  `5` = "Very healthy"
))

# reorder factor levels

responses$Healthy <- factor(responses$Healthy,levels = c("Very healthy", "4", "3", "2", "Not very healt[illegible]

# Natural

responses <- responses %>% mutate(Natural = recode(Natural,
  `1` = "Not very natural",
  `2` = "2",
  `3` = "3",
  `4` = "4",
  `5` = "Very natural"
))

# reorder factor levels

responses$Natural <- factor(responses$Natural,levels = c("Very natural", "4", "3", "2", "Not very natur[illegible]

# Sustainable

responses <- responses %>% mutate(Sustainable = recode(Sustainable,
  `1` = "Not very sustainable",
  `2` = "2",
  `3` = "3",
  `4` = "4",
  `5` = "Very sustainable"
))

# reorder factor levels

responses$Sustainable <- factor(responses$Sustainable,levels = c("Very sustainable", "4", "3", "2", "No[illegible]

# Satiating
responses <- responses %>% mutate(Satiating = recode(Satiating,
  `1` = "Not very satiating",
  `2` = "2",
  `3` = "3",
  `4` = "4",
  `5` = "Very satiating"
))

# reorder factor levels

responses$Satiating <- factor(responses$Satiating,levels = c("Very satiating", "4", "3", "2", "Not very [illegible]
```

```
(plot_4d <- responses %>%
  drop_na(c(Dish, Like)) %>%
  ggplot(aes(x = Dish, fill = Like)) +
  scale_fill_manual(values = c("#26315d", "#374783", "#a6cfe5", "#fe6f61", "#f4e79e"), drop = FALSE) +
  geom_bar(position = "fill") +
```

```r
  theme_minimal() +
  theme(
    axis.title.y = element_text(angle = 0, vjust = 0.5, hjust = 1),
    axis.text.x = element_text(angle = 45, vjust = 1, hjust = 1)
  ) +
  labs(
    title = "",
    x = "Dish",
    y = "Responses"
  ))
```

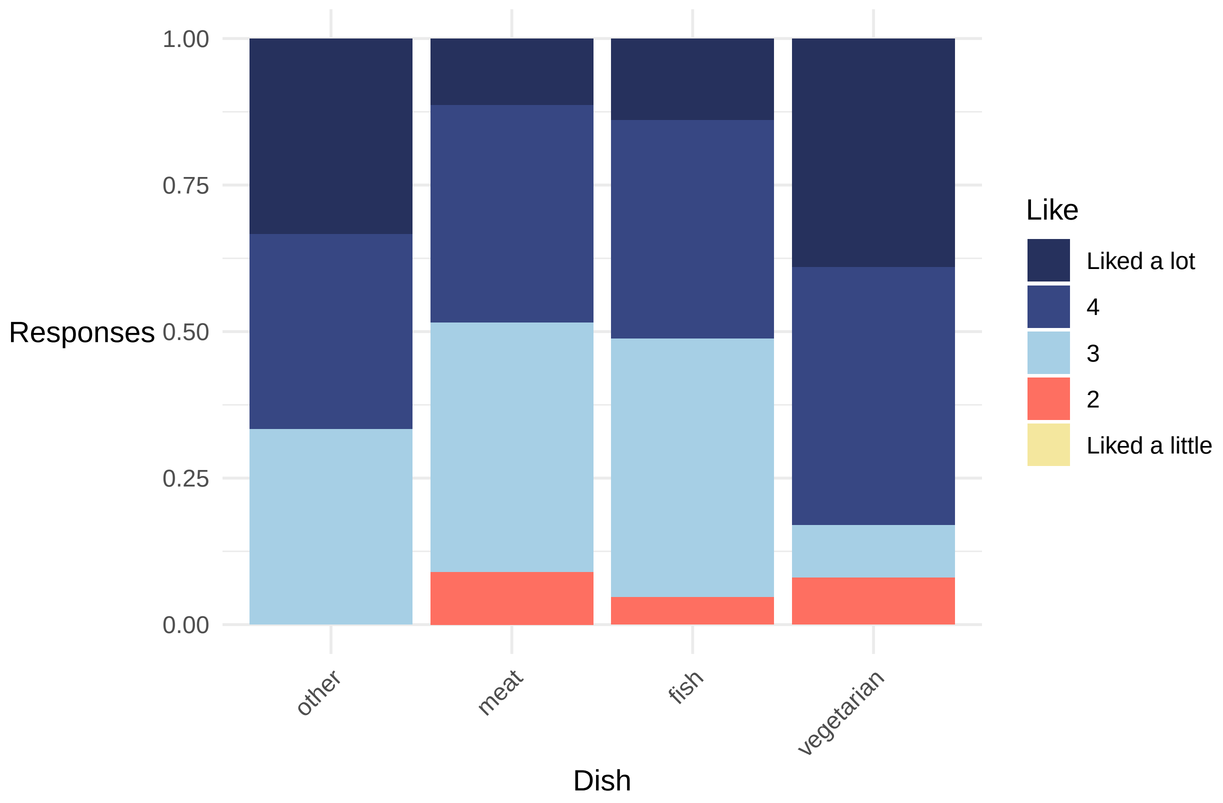


```r
responses %>%
  count(Dish, Like, .drop = FALSE) %>%
  group_by(Dish) %>%
  mutate(prop = n/sum(n)) %>% knitr::kable()
```

Dish	Like	n	prop
other	Liked a lot	1	0.2000
other	4	1	0.2000
other	3	1	0.2000
other	2	0	0.0000
other	Liked a little	0	0.0000
other	NA	2	0.4000

Dish	Like	n	prop
meat	Liked a lot	19	0.1011
meat	4	62	0.3298
meat	3	71	0.3777
meat	2	15	0.0798
meat	Liked a little	0	0.0000
meat	NA	21	0.1117
fish	Liked a lot	6	0.1224
fish	4	16	0.3265
fish	3	19	0.3878
fish	2	2	0.0408
fish	Liked a little	0	0.0000
fish	NA	6	0.1224
vegetarian	Liked a lot	39	0.3451
vegetarian	4	44	0.3894
vegetarian	3	9	0.0796
vegetarian	2	8	0.0708
vegetarian	Liked a little	0	0.0000
vegetarian	NA	13	0.1150
NA	Liked a lot	0	0.0000
NA	4	0	0.0000
NA	3	0	0.0000
NA	2	0	0.0000
NA	Liked a little	0	0.0000
NA	NA	15	1.0000

```
(plot_5<- responses %>%
  drop_na(c(Dish, Sustainable)) %>%
  ggplot(aes(x = Dish, fill = Sustainable)) +
  scale_fill_manual(values = c("#26315d", "#374783", "#a6cfe5", "#fe6f61", "#f4e79e"), drop = FALSE) +
  geom_bar(position = "fill") +
  theme_minimal() +
  theme(
    axis.title.y = element_text(angle = 0, vjust = 0.5, hjust = 1),
    axis.text.x = element_text(angle = 45, vjust = 1, hjust = 1)
  ) +
  labs(
    title = "",
    x = "Dish",
    y = "Responses"
  ))
```

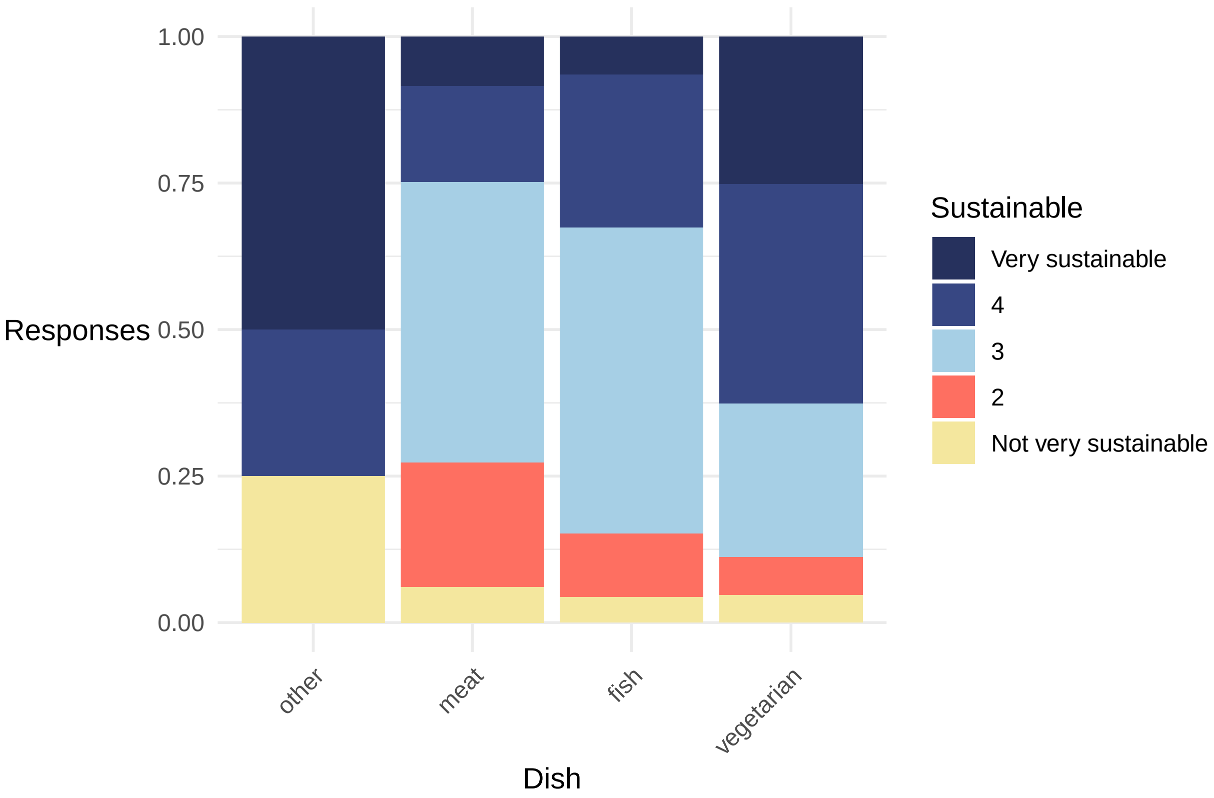


```
responses %>%
  count(Dish, Sustainable, .drop = FALSE) %>%
  group_by(Dish) %>%
  mutate(prop = n/sum(n)) %>% knitr::kable()
```

Dish	Sustainable	n	prop
other	Very sustainable	2	0.4000
other	4	1	0.2000
other	3	0	0.0000
other	2	0	0.0000
other	Not very sustainable	1	0.2000
other	NA	1	0.2000
meat	Very sustainable	14	0.0745
meat	4	27	0.1436
meat	3	79	0.4202
meat	2	35	0.1862
meat	Not very sustainable	10	0.0532
meat	NA	23	0.1223
fish	Very sustainable	3	0.0612
fish	4	12	0.2449
fish	3	24	0.4898
fish	2	5	0.1020
fish	Not very sustainable	2	0.0408
fish	NA	3	0.0612

Dish	Sustainable	n	prop
vegetarian	Very sustainable	27	0.2389
vegetarian	4	40	0.3540
vegetarian	3	28	0.2478
vegetarian	2	7	0.0619
vegetarian	Not very sustainable	5	0.0442
vegetarian	NA	6	0.0531
NA	Very sustainable	0	0.0000
NA	4	0	0.0000
NA	3	0	0.0000
NA	2	0	0.0000
NA	Not very sustainable	0	0.0000
NA	NA	15	1.0000

```
(plot_6 <- responses %>%
  drop_na(c(Dish, Healthy)) %>%
  ggplot(aes(x = Dish, fill = Healthy)) +
  scale_fill_manual(values = c("#26315d", "#374783", "#a6cfe5", "#fe6f61", "#f4e79e"), drop = FALSE) +
  geom_bar(position = "fill") +
  theme_minimal() +
  theme(
    axis.title.y = element_text(angle = 0, vjust = 0.5, hjust = 1),
    axis.text.x = element_text(angle = 45, vjust = 1, hjust = 1)
  ) +
  labs(
    title = "",
    x = "Dish",
    y = "Responses"
  ))
```

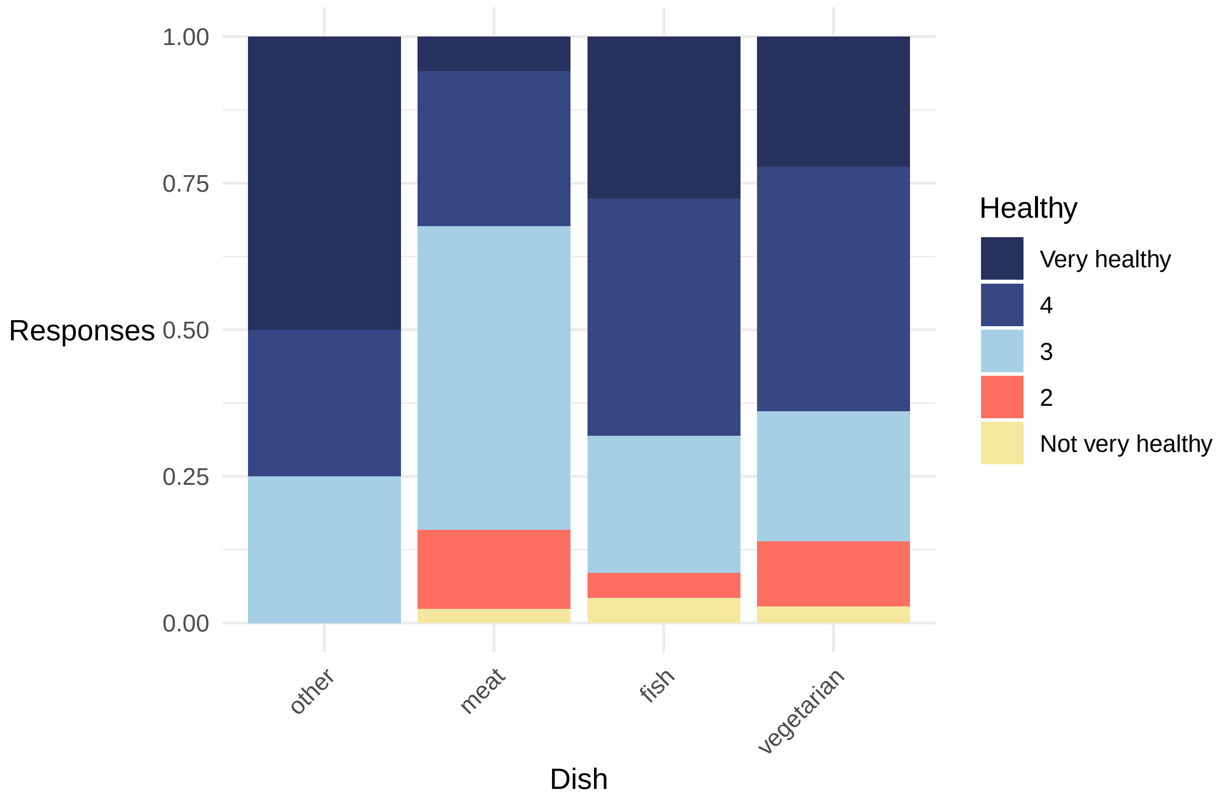


```
responses %>%
  count(Dish, Healthy, .drop = FALSE) %>%
  group_by(Dish) %>%
  mutate(prop = n/sum(n)) %>% knitr::kable()
```

Dish	Healthy	n	prop
other	Very healthy	2	0.4000
other	4	1	0.2000
other	3	1	0.2000
other	2	0	0.0000
other	Not very healthy	0	0.0000
other	NA	1	0.2000
meat	Very healthy	10	0.0532
meat	4	45	0.2394
meat	3	88	0.4681
meat	2	23	0.1223
meat	Not very healthy	4	0.0213
meat	NA	18	0.0957
fish	Very healthy	13	0.2653
fish	4	19	0.3878
fish	3	11	0.2245
fish	2	2	0.0408
fish	Not very healthy	2	0.0408
fish	NA	2	0.0408

Dish	Healthy	n	prop
vegetarian	Very healthy	24	0.2124
vegetarian	4	45	0.3982
vegetarian	3	24	0.2124
vegetarian	2	12	0.1062
vegetarian	Not very healthy	3	0.0265
vegetarian	NA	5	0.0442
NA	Very healthy	0	0.0000
NA	4	0	0.0000
NA	3	0	0.0000
NA	2	0	0.0000
NA	Not very healthy	0	0.0000
NA	NA	15	1.0000

```
(plot_7 <- responses %>%
  drop_na(c(Dish, Natural)) %>%
  ggplot(aes(x = Dish, fill = Natural)) +
  scale_fill_manual(values = c("#26315d", "#374783", "#a6cfe5", "#fe6f61", "#f4e79e"), drop = FALSE) +
  geom_bar(position = "fill") +
  theme_minimal() +
  theme(
    axis.title.y = element_text(angle = 0, vjust = 0.5, hjust = 1),
    axis.text.x = element_text(angle = 45, vjust = 1, hjust = 1)
  ) +
  labs(
    title = "",
    x = "Dish",
    y = "Responses"
  ))
```

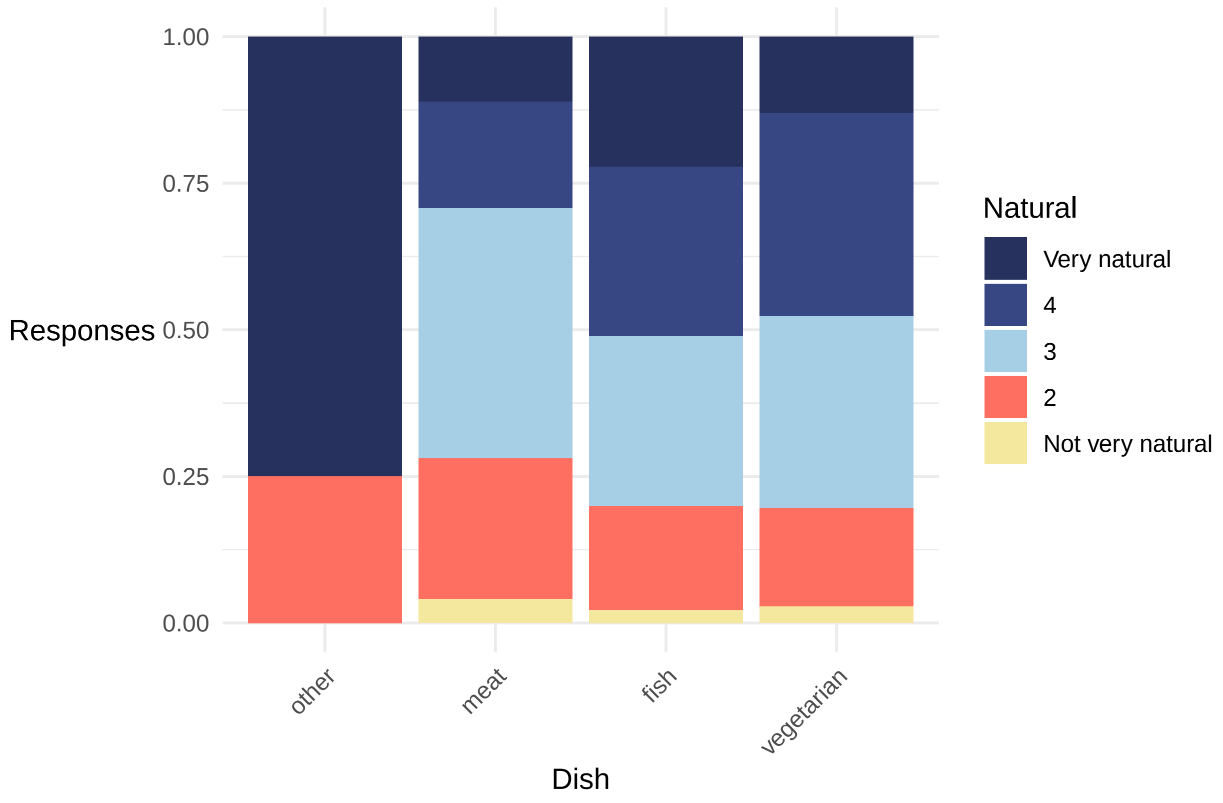


```
responses %>%
  count(Dish, Natural, .drop = FALSE) %>%
  group_by(Dish) %>%
  mutate(prop = n/sum(n)) %>% knitr::kable()
```

Dish	Natural	n	prop
other	Very natural	3	0.6000
other	4	0	0.0000
other	3	0	0.0000
other	2	1	0.2000
other	Not very natural	0	0.0000
other	NA	1	0.2000
meat	Very natural	19	0.1011
meat	4	31	0.1649
meat	3	73	0.3883
meat	2	41	0.2181
meat	Not very natural	7	0.0372
meat	NA	17	0.0904
fish	Very natural	10	0.2041
fish	4	13	0.2653
fish	3	13	0.2653
fish	2	8	0.1633
fish	Not very natural	1	0.0204
fish	NA	4	0.0816

Dish	Natural	n	prop
vegetarian	Very natural	14	0.1239
vegetarian	4	37	0.3274
vegetarian	3	35	0.3097
vegetarian	2	18	0.1593
vegetarian	Not very natural	3	0.0265
vegetarian	NA	6	0.0531
NA	Very natural	0	0.0000
NA	4	0	0.0000
NA	3	0	0.0000
NA	2	0	0.0000
NA	Not very natural	0	0.0000
NA	NA	15	1.0000

```
(plot_8 <- responses %>%
  drop_na(c(Dish, Satiating)) %>%
  ggplot(aes(x = Dish, fill = Satiating)) +
  scale_fill_manual(values = c("#26315d", "#374783", "#a6cfe5", "#fe6f61", "#f4e79e"), drop = FALSE) +
  geom_bar(position = "fill") +
  theme_minimal() +
  theme(
    axis.title.y = element_text(angle = 0, vjust = 0.5, hjust = 1),
    axis.text.x = element_text(angle = 45, vjust = 1, hjust = 1)
  ) +
  labs(
    title = "",
    x = "Dish",
    y = "Responses"
  ))
```

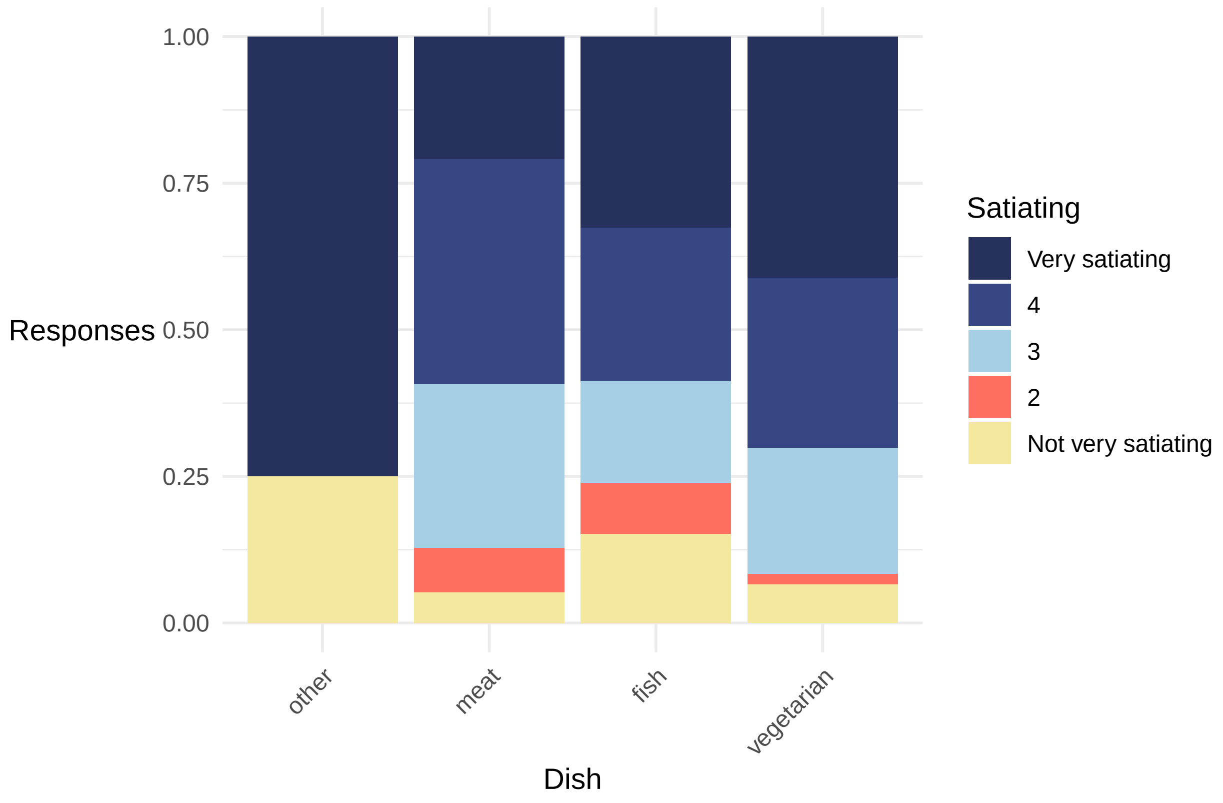


```
responses %>%
  count(Dish, Satiating, .drop = FALSE) %>%
  group_by(Dish) %>%
  mutate(prop = n/sum(n)) %>% knitr::kable()
```

Dish	Satiating	n	prop
other	Very satiating	3	0.6000
other	4	0	0.0000
other	3	0	0.0000
other	2	0	0.0000
other	Not very satiating	1	0.2000
other	NA	1	0.2000
meat	Very satiating	36	0.1915
meat	4	66	0.3511
meat	3	48	0.2553
meat	2	13	0.0691
meat	Not very satiating	9	0.0479
meat	NA	16	0.0851
fish	Very satiating	15	0.3061
fish	4	12	0.2449
fish	3	8	0.1633
fish	2	4	0.0816
fish	Not very satiating	7	0.1429
fish	NA	3	0.0612

Dish	Satiating	n	prop
vegetarian	Very satiating	44	0.3894
vegetarian	4	31	0.2743
vegetarian	3	23	0.2035
vegetarian	2	2	0.0177
vegetarian	Not very satiating	7	0.0619
vegetarian	NA	6	0.0531
NA	Very satiating	0	0.0000
NA	4	0	0.0000
NA	3	0	0.0000
NA	2	0	0.0000
NA	Not very satiating	0	0.0000
NA	NA	15	1.0000

Inferential

```
rated_vars <- c("Like", "Sustainable", "Healthy", "Natural", "Satiating")

responses_inf <- responses %>%
  dplyr::filter(
    !is.na(Dish),
    Dish %in% c("meat", "fish", "vegetarian")
  ) %>%
  dplyr::mutate(
    # make vegetarian the reference category
    Dish = forcats::fct_rev(Dish),
    dplyr::across(
      .cols = all_of(rated_vars),
      .fns = ~ forcats::fct_rev(.x),
    ),
    dplyr::across(
      .cols = all_of(rated_vars),
      .fns = ~ as.numeric(.x),
      .names = "{.col}_num"
    )
  )
```

Means and standard deviations

```
responses_inf %>%
  dplyr::group_by(Dish) %>%
  dplyr::summarise(
    dplyr::across(
      .cols = dplyr::ends_with("_num"),
      .fns = list(
        mean = ~ mean(.x, na.rm = TRUE),
        sd = ~ sd(.x, na.rm = TRUE)
      )
    )
  ) %>%
  dplyr::rename_with(
```

```r
    .cols = -Dish,
    .fn = ~ stringr::str_remove(.x, "_num")
  ) %>%
  tibble::column_to_rownames(var = "Dish") %>%
  t() %>%
  knitr::kable()
```

	vegetarian	fish	meat
Like_mean	4.1400	3.6047	3.5090
Like_sd	0.8879	0.7910	0.8131
Sustainable_mean	3.7196	3.1957	3.0000
Sustainable_sd	1.0622	0.8849	0.9815
Healthy_mean	3.6944	3.8298	3.2000
Healthy_sd	1.0273	1.0283	0.8328
Natural_mean	3.3832	3.5111	3.0819
Natural_sd	1.0061	1.1000	1.0142
Satiating_mean	3.9626	3.5217	3.6221
Satiating_sd	1.1404	1.4258	1.0608

Location-scale ordinal models and relevant contrasts

```r
model_Like <- ordinal::clm(
  Like ~ Dish,
  scale = ~ Dish,
  data = responses_inf, link = "probit"
)

model_Like %>% summary()
```

```
## formula: Like ~ Dish
## scale:   ~Dish
## data:    responses_inf
##
##  link   threshold nobs logLik  AIC    niter max.grad cond.H
##  probit flexible  310  -370.00 754.01 7(0)  3.24e-11 4.5e+01
##
## Coefficients:
##          Estimate Std. Error z value Pr(>|z|)
## Dishfish   -0.618      0.165   -3.75  0.00018 ***
## Dishmeat   -0.712      0.135   -5.26  1.4e-07 ***
## ---
## Signif. codes:  0 '***' 0.001 '**' 0.01 '*' 0.05 '.' 0.1 ' ' 1
##
## log-scale coefficients:
##          Estimate Std. Error z value Pr(>|z|)
## Dishfish   -0.349      0.176   -1.99    0.047 *
## Dishmeat   -0.301      0.132   -2.28    0.023 *
## ---
## Signif. codes:  0 '***' 0.001 '**' 0.01 '*' 0.05 '.' 0.1 ' ' 1
##
## Threshold coefficients:
```

```
##                Estimate Std. Error z value
## 2|3              -1.659      0.194   -8.58
## 3|4              -0.725      0.126   -5.76
## 4|Liked a lot     0.222      0.124    1.80
## (40 observations deleted due to missingness)
```

```
contrasts_Like <- model_Like %>%
  emmeans::emmeans(specs = ~ Dish) %>% pairs()

contrasts_Like %>% knitr::kable()
```

contrast	estimate	SE	df	z.ratio	p.value
vegetarian - fish	0.6175	0.1646	Inf	3.7511	0.0005
vegetarian - meat	0.7116	0.1352	Inf	5.2631	0.0000
fish - meat	0.0941	0.1318	Inf	0.7139	0.7552

```
model_Sustainable <- ordinal::clm(
  Sustainable ~ Dish,
  scale = ~ Dish,
  data = responses_inf, link = "probit"
)

model_Sustainable %>% summary()
```

```
## formula: Sustainable ~ Dish
## scale:   ~Dish
## data:    responses_inf
##
##  link   threshold nobs logLik  AIC    niter max.grad cond.H
##  probit flexible  318  -434.29 884.58 8(0)  5.68e-14 4.3e+01
##
## Coefficients:
##          Estimate Std. Error z value Pr(>|z|)
## Dishfish   -0.518      0.160   -3.24   0.0012 **
## Dishmeat   -0.697      0.134   -5.21  1.8e-07 ***
## ---
## Signif. codes:  0 '***' 0.001 '**' 0.01 '*' 0.05 '.' 0.1 ' ' 1
##
## log-scale coefficients:
##          Estimate Std. Error z value Pr(>|z|)
## Dishfish   -0.292      0.151   -1.93    0.053 .
## Dishmeat   -0.164      0.112   -1.46    0.145
## ---
## Signif. codes:  0 '***' 0.001 '**' 0.01 '*' 0.05 '.' 0.1 ' ' 1
##
## Threshold coefficients:
##                         Estimate Std. Error z value
## Not very sustainable|2   -1.915      0.201   -9.55
## 2|3                      -1.228      0.147   -8.33
## 3|4                      -0.189      0.110   -1.72
## 4|Very sustainable        0.589      0.125    4.73
## (32 observations deleted due to missingness)
```

```r
contrasts_Sustainable <- model_Sustainable %>%
  emmeans::emmeans(specs = ~ Dish) %>% pairs()

contrasts_Sustainable %>% knitr::kable()
```

contrast	estimate	SE	df	z.ratio	p.value
vegetarian - fish	0.5176	0.1597	Inf	3.241	0.0034
vegetarian - meat	0.6970	0.1337	Inf	5.215	0.0000
fish - meat	0.1793	0.1378	Inf	1.301	0.3945

```r
model_Healthy <- ordinal::clm(
  Healthy ~ Dish,
  scale = ~ Dish,
  data = responses_inf, link = "probit"
)

model_Healthy %>% summary()
```

```
## formula: Healthy ~ Dish
## scale:   ~Dish
## data:    responses_inf
##
##  link   threshold nobs logLik  AIC    niter max.grad cond.H
##  probit flexible  325  -422.28 860.57 8(0)  2.51e-13 4.1e+01
##
## Coefficients:
##          Estimate Std. Error z value Pr(>|z|)
## Dishfish    0.153      0.194    0.79     0.43
## Dishmeat   -0.519      0.123   -4.23  2.3e-05 ***
## ---
## Signif. codes:  0 '***' 0.001 '**' 0.01 '*' 0.05 '.' 0.1 ' ' 1
##
## log-scale coefficients:
##          Estimate Std. Error z value Pr(>|z|)
## Dishfish   0.0334     0.1636     0.2   0.8380
## Dishmeat  -0.3231     0.1077    -3.0   0.0027 **
## ---
## Signif. codes:  0 '***' 0.001 '**' 0.01 '*' 0.05 '.' 0.1 ' ' 1
##
## Threshold coefficients:
##                    Estimate Std. Error z value
## Not very healthy|2   -1.913      0.200   -9.56
## 2|3                  -1.208      0.143   -8.46
## 3|4                  -0.246      0.109   -2.25
## 4|Very healthy        0.715      0.128    5.59
## (25 observations deleted due to missingness)
```

```r
contrasts_Healthy <- model_Healthy %>%
  emmeans::emmeans(specs = ~ Dish) %>% pairs()

contrasts_Healthy %>% knitr::kable()
```

contrast	estimate	SE	df	z.ratio	p.value
vegetarian - fish	-0.1530	0.1941	Inf	-0.7884	0.7102
vegetarian - meat	0.5187	0.1226	Inf	4.2297	0.0001
fish - meat	0.6717	0.1834	Inf	3.6627	0.0007

```r
model_Natural <- ordinal::clm(
  Natural ~ Dish,
  scale = ~ Dish,
  data = responses_inf, link = "probit"
)

model_Natural %>% summary()
```

```
## formula: Natural ~ Dish
## scale:   ~Dish
## data:    responses_inf
##
##  link   threshold nobs logLik  AIC    niter max.grad cond.H
##  probit flexible  323  -455.26 926.52 6(0)  1.52e-07 3.1e+01
##
## Coefficients:
##          Estimate Std. Error z value Pr(>|z|)
## Dishfish    0.155      0.208    0.74    0.458
## Dishmeat   -0.309      0.132   -2.34    0.019 *
## ---
## Signif. codes:  0 '***' 0.001 '**' 0.01 '*' 0.05 '.' 0.1 ' ' 1
##
## log-scale coefficients:
##          Estimate Std. Error z value Pr(>|z|)
## Dishfish   0.1371     0.1581    0.87     0.39
## Dishmeat   0.0197     0.1048    0.19     0.85
##
## Threshold coefficients:
##                    Estimate Std. Error z value
## Not very natural|2   -2.042      0.216   -9.47
## 2|3                  -0.880      0.129   -6.84
## 3|4                   0.164      0.110    1.49
## 4|Very natural        1.023      0.139    7.37
## (27 observations deleted due to missingness)
```

```r
contrasts_Natural <- model_Natural %>%
  emmeans::emmeans(specs = ~ Dish) %>% pairs()

contrasts_Natural %>% knitr::kable()
```

contrast	estimate	SE	df	z.ratio	p.value
vegetarian - fish	-0.1547	0.2084	Inf	-0.7425	0.7381
vegetarian - meat	0.3092	0.1323	Inf	2.3363	0.0509
fish - meat	0.4639	0.2025	Inf	2.2908	0.0571

```r
model_Satiating <- ordinal::clm(
  Satiating ~ Dish,
  scale = ~ Dish,
  data = responses_inf, link = "probit"
)

model_Satiating %>% summary()
```

```
## formula: Satiating ~ Dish
## scale:   ~Dish
## data:    responses_inf
##
##  link   threshold nobs logLik  AIC    niter max.grad cond.H
##  probit flexible  325  -452.53 921.07 8(1)  1.02e-08 5.8e+01
##
## Coefficients:
##          Estimate Std. Error z value Pr(>|z|)
## Dishfish   -0.322      0.224   -1.44   0.1503
## Dishmeat   -0.382      0.122   -3.12   0.0018 **
## ---
## Signif. codes:  0 '***' 0.001 '**' 0.01 '*' 0.05 '.' 0.1 ' ' 1
##
## log-scale coefficients:
##          Estimate Std. Error z value Pr(>|z|)
## Dishfish    0.206      0.199    1.04    0.300
## Dishmeat   -0.287      0.129   -2.23    0.026 *
## ---
## Signif. codes:  0 '***' 0.001 '**' 0.01 '*' 0.05 '.' 0.1 ' ' 1
##
## Threshold coefficients:
##                        Estimate Std. Error z value
## Not very satiating|2   -1.573      0.180   -8.74
## 2|3                    -1.261      0.153   -8.27
## 3|4                    -0.553      0.114   -4.85
## 4|Very satiating        0.225      0.121    1.86
## (25 observations deleted due to missingness)
```

```r
contrasts_Satiating <- model_Satiating %>%
  emmeans::emmeans(specs = ~ Dish) %>% pairs()

contrasts_Satiating %>% knitr::kable()
```

contrast	estimate	SE	df	z.ratio	p.value
vegetarian - fish	0.3223	0.2241	Inf	1.4384	0.3212
vegetarian - meat	0.3817	0.1223	Inf	3.1225	0.0051
fish - meat	0.0594	0.2070	Inf	0.2871	0.9556

```r
sessioninfo::session_info()
```

```
## - Session info ---------------------------------------------------------------
```

```
##  setting  value
##  version  R version 4.2.1 (2022-06-23 ucrt)
##  os       Windows 10 x64 (build 19044)
##  system   x86_64, mingw32
##  ui       RTerm
##  language (EN)
##  collate  English_United States.utf8
##  ctype    English_United States.utf8
##  tz       Europe/London
##  date     2022-10-28
##  pandoc   2.18 @ C:/Program Files/RStudio/bin/quarto/bin/tools/ (via rmarkdown)
##
## - Packages -------------------------------------------------------------------
##  package       * version    date (UTC) lib source
##  abind           1.4-5      2016-07-21 [1] CRAN (R 4.2.0)
##  assertthat      0.2.1      2019-03-21 [1] CRAN (R 4.2.1)
##  backports       1.4.1      2021-12-13 [1] CRAN (R 4.2.0)
##  broom           1.0.0      2022-07-01 [1] CRAN (R 4.2.1)
##  broom.helpers   1.8.0      2022-07-05 [1] CRAN (R 4.2.1)
##  car             3.1-0      2022-06-15 [1] CRAN (R 4.2.1)
##  carData         3.0-5      2022-01-06 [1] CRAN (R 4.2.1)
##  cellranger      1.1.0      2016-07-27 [1] CRAN (R 4.2.1)
##  checkmate       2.1.0      2022-04-21 [1] CRAN (R 4.2.1)
##  cli             3.3.0      2022-04-25 [1] CRAN (R 4.2.1)
##  coda            0.19-4     2020-09-30 [1] CRAN (R 4.2.1)
##  codetools       0.2-18     2020-11-04 [2] CRAN (R 4.2.1)
##  colorspace      2.0-3      2022-02-21 [1] CRAN (R 4.2.1)
##  crayon          1.5.1      2022-03-26 [1] CRAN (R 4.2.1)
##  DBI             1.1.3      2022-06-18 [1] CRAN (R 4.2.1)
##  dbplyr          2.2.1      2022-06-27 [1] CRAN (R 4.2.1)
##  digest          0.6.29     2021-12-01 [1] CRAN (R 4.2.1)
##  dplyr         * 1.0.9      2022-04-28 [1] CRAN (R 4.2.1)
##  ellipsis        0.3.2      2021-04-29 [1] CRAN (R 4.2.1)
##  emmeans         1.7.5      2022-06-22 [1] CRAN (R 4.2.1)
##  estimability    1.4        2022-07-03 [1] CRAN (R 4.2.1)
##  evaluate        0.15       2022-02-18 [1] CRAN (R 4.2.1)
##  fansi           1.0.3      2022-03-24 [1] CRAN (R 4.2.1)
##  farver          2.1.1      2022-07-06 [1] CRAN (R 4.2.1)
##  fastmap         1.1.0      2021-01-25 [1] CRAN (R 4.2.1)
##  forcats       * 0.5.1      2021-01-27 [1] CRAN (R 4.2.1)
##  fs              1.5.2      2021-12-08 [1] CRAN (R 4.2.1)
##  gargle          1.2.0      2021-07-02 [1] CRAN (R 4.2.1)
##  generics        0.1.3      2022-07-05 [1] CRAN (R 4.2.1)
##  ggplot2       * 3.3.6      2022-05-03 [1] CRAN (R 4.2.1)
##  ggpubr        * 0.4.0      2020-06-27 [1] CRAN (R 4.2.1)
##  ggsignif        0.6.3      2021-09-09 [1] CRAN (R 4.2.1)
##  glue            1.6.2      2022-02-24 [1] CRAN (R 4.2.1)
##  googledrive     2.0.0      2021-07-08 [1] CRAN (R 4.2.1)
##  googlesheets4   1.0.0      2021-07-21 [1] CRAN (R 4.2.1)
##  gt            * 0.6.0      2022-05-24 [1] CRAN (R 4.2.1)
##  gtable          0.3.0      2019-03-25 [1] CRAN (R 4.2.1)
##  gtsummary     * 1.6.1      2022-06-22 [1] CRAN (R 4.2.1)
##  haven           2.5.0      2022-04-15 [1] CRAN (R 4.2.1)
##  here          * 1.0.1      2020-12-13 [1] CRAN (R 4.2.1)
```

```
##  highr           0.9        2021-04-16 [1] CRAN (R 4.2.1)
##  hms             1.1.1      2021-09-26 [1] CRAN (R 4.2.1)
##  htmltools       0.5.3      2022-07-18 [1] CRAN (R 4.2.1)
##  httr            1.4.3      2022-05-04 [1] CRAN (R 4.2.1)
##  jsonlite        1.8.0      2022-02-22 [1] CRAN (R 4.2.1)
##  knitr         * 1.39       2022-04-26 [1] CRAN (R 4.2.1)
##  labeling        0.4.2      2020-10-20 [1] CRAN (R 4.2.0)
##  lattice         0.20-45    2021-09-22 [2] CRAN (R 4.2.1)
##  lifecycle       1.0.1      2021-09-24 [1] CRAN (R 4.2.1)
##  lubridate       1.8.0      2021-10-07 [1] CRAN (R 4.2.1)
##  magrittr        2.0.3      2022-03-30 [1] CRAN (R 4.2.1)
##  MASS            7.3-58     2022-07-14 [1] CRAN (R 4.2.1)
##  Matrix          1.5-1      2022-09-13 [1] CRAN (R 4.2.1)
##  modelr          0.1.8      2020-05-19 [1] CRAN (R 4.2.1)
##  multcomp        1.4-19     2022-04-26 [1] CRAN (R 4.2.1)
##  munsell         0.5.0      2018-06-12 [1] CRAN (R 4.2.1)
##  mvtnorm         1.1-3      2021-10-08 [1] CRAN (R 4.2.0)
##  numDeriv        2016.8-1.1 2019-06-06 [1] CRAN (R 4.2.0)
##  ordinal         2019.12-10 2019-12-15 [1] CRAN (R 4.2.1)
##  pillar          1.8.0      2022-07-18 [1] CRAN (R 4.2.1)
##  pkgconfig       2.0.3      2019-09-22 [1] CRAN (R 4.2.1)
##  purrr         * 0.3.4      2020-04-17 [1] CRAN (R 4.2.1)
##  R6              2.5.1      2021-08-19 [1] CRAN (R 4.2.1)
##  readr         * 2.1.2      2022-01-30 [1] CRAN (R 4.2.1)
##  readxl        * 1.4.0      2022-03-28 [1] CRAN (R 4.2.1)
##  reprex          2.0.1      2021-08-05 [1] CRAN (R 4.2.1)
##  rlang           1.0.4      2022-07-12 [1] CRAN (R 4.2.1)
##  rmarkdown       2.14       2022-04-25 [1] CRAN (R 4.2.1)
##  rprojroot       2.0.3      2022-04-02 [1] CRAN (R 4.2.1)
##  rstatix         0.7.0      2021-02-13 [1] CRAN (R 4.2.1)
##  rstudioapi      0.13       2020-11-12 [1] CRAN (R 4.2.1)
##  rvest           1.0.2      2021-10-16 [1] CRAN (R 4.2.1)
##  sandwich        3.0-2      2022-06-15 [1] CRAN (R 4.2.1)
##  scales          1.2.0      2022-04-13 [1] CRAN (R 4.2.1)
##  sessioninfo   * 1.2.2      2021-12-06 [1] CRAN (R 4.2.1)
##  stringi         1.7.8      2022-07-11 [1] CRAN (R 4.2.1)
##  stringr       * 1.4.0      2019-02-10 [1] CRAN (R 4.2.1)
##  survival        3.3-1      2022-03-03 [2] CRAN (R 4.2.1)
##  TH.data         1.1-1      2022-04-26 [1] CRAN (R 4.2.1)
##  tibble        * 3.1.8      2022-07-22 [1] CRAN (R 4.2.1)
##  tidyr         * 1.2.0      2022-02-01 [1] CRAN (R 4.2.1)
##  tidyselect      1.1.2      2022-02-21 [1] CRAN (R 4.2.1)
##  tidyverse     * 1.3.2      2022-07-18 [1] CRAN (R 4.2.1)
##  tzdb            0.3.0      2022-03-28 [1] CRAN (R 4.2.1)
##  ucminf          1.1-4      2016-08-18 [1] CRAN (R 4.2.0)
##  utf8            1.2.2      2021-07-24 [1] CRAN (R 4.2.1)
##  vctrs           0.4.1      2022-04-13 [1] CRAN (R 4.2.1)
##  withr           2.5.0      2022-03-03 [1] CRAN (R 4.2.1)
##  xfun            0.31       2022-05-10 [1] CRAN (R 4.2.1)
##  xml2            1.3.3      2021-11-30 [1] CRAN (R 4.2.1)
##  xtable          1.8-4      2019-04-21 [1] CRAN (R 4.2.1)
##  yaml            2.3.5      2022-02-21 [1] CRAN (R 4.2.0)
##  zoo             1.8-10     2022-04-15 [1] CRAN (R 4.2.1)
##
```

```
##  [1] C:/Users/admin/AppData/Local/R/win-library/4.2
##  [2] C:/Program Files/R/R-4.2.1/library
##
## -----------------------------------------------------------------------------
```
