# Supplementary material for: Promoting plant-based eating in meat-centric meal contexts: a field study
Source: Public Health Nutr. 2023 Sep 15;26(11):2619–27. doi: 10.1017/S1368980023001763 (PMC10641616; doi:10.1017/S1368980023001763)
Supplement: Supplementary file 1 [file S1368980023001763sup.zip › S1368980023001763sup002.pdf]

Intervention Analysis

SUSTAINMEALS

Document last updated: 2022-10-13 13:12:17

```
library(here)
library(tidyverse)
library(sessioninfo)
library(lme4)
library(emmeans)
library(gtsummary)
library(knitr)
library(broom.mixed)

options(digits = 3)
```

```
data_final <- readRDS(here::here("01_wrangling", "data_final.RDS"))
data_intervention <- readRDS(here::here("01_wrangling", "data_intervention.RDS"))
```

Descriptives

Using all the data

Meals sold by type and time (absolute numbers)

```
data_final %>%
  ggplot(aes(x = date, y = sold, fill = type)) +
  geom_col(width = 1) +
  facet_grid(meal ~ ., scales = "free_y")
```

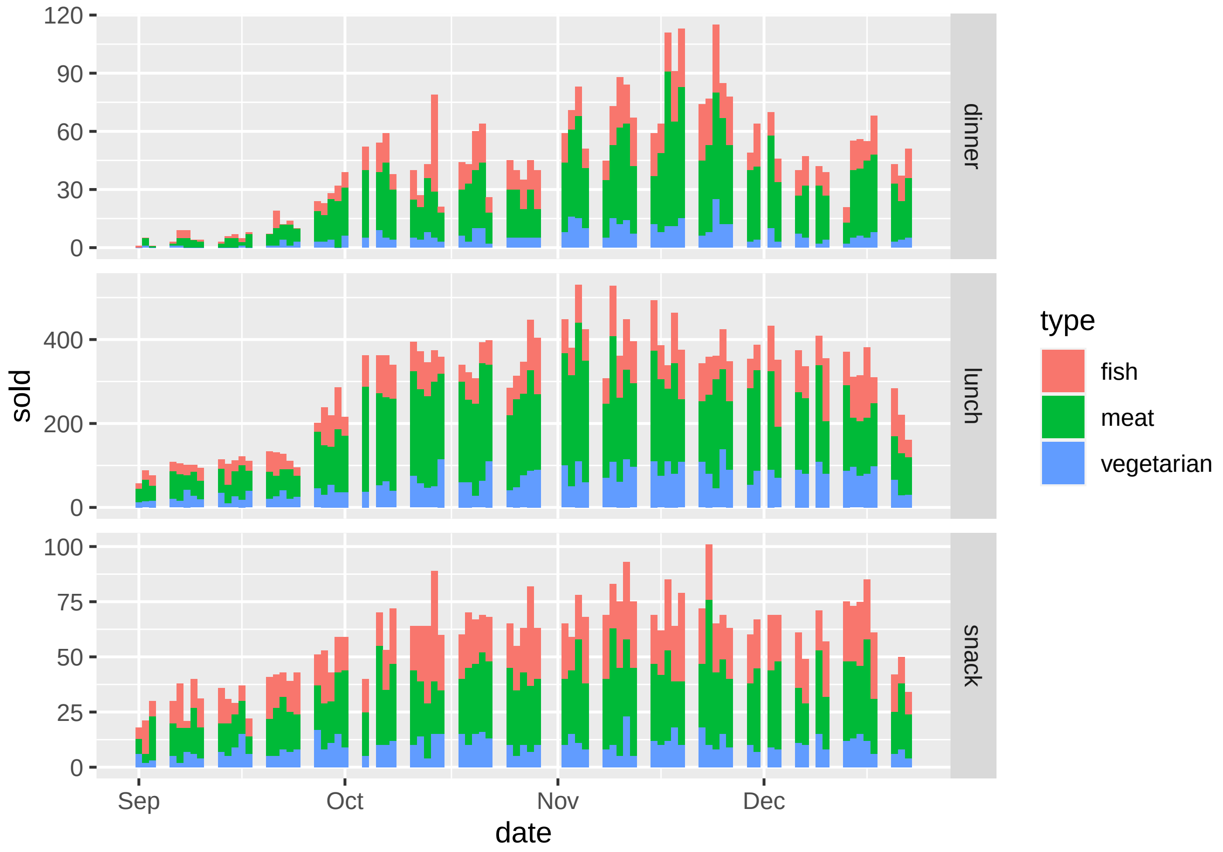

Meals sold by type and time (percentages)

```
data_final %>%
  ggplot(aes(x = date, y = prop_sold, fill = type)) +
  geom_col(width = 1) +
  facet_grid(meal ~ .)
```

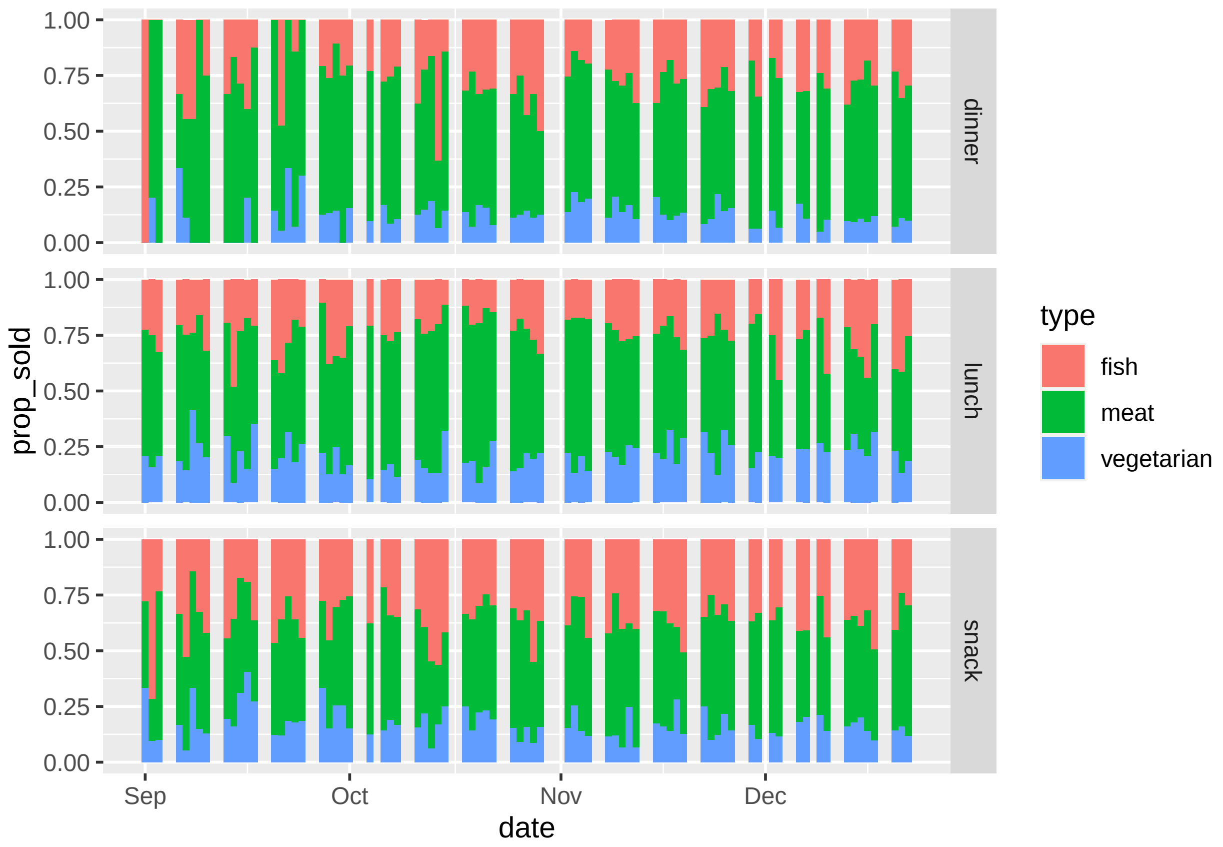

Tables

```
data_final %>%
  group_by(type) %>%
  summarise(
    total = sum(sold, na.rm = TRUE),
    prop = sum(sold, na.rm = TRUE)/sum(total_sold, na.rm = TRUE)
  ) %>% knitr::kable()
```

type	total	prop
fish	8072	0.262
meat	16836	0.546
vegetarian	5931	0.192

```
data_final %>%
  group_by(meal, type) %>%
  summarise(
    total = sum(sold, na.rm = TRUE),
    prop = sum(sold, na.rm = TRUE)/sum(total_sold, na.rm = TRUE)
  ) %>% knitr::kable()
```

meal	type	total	prop
dinner	fish	943	0.277
dinner	meat	2024	0.595
dinner	vegetarian	432	0.127
lunch	fish	5535	0.241
lunch	meat	12666	0.552
lunch	vegetarian	4752	0.207
snack	fish	1594	0.355
snack	meat	2146	0.478
snack	vegetarian	747	0.166

Only November and December

Meals sold by type and time (absolute numbers)

```
data_intervention %>%
  ggplot(aes(x = date, y = prop_sold, fill = type)) +
  geom_col(width = 1) +
  facet_grid(meal ~ .)
```

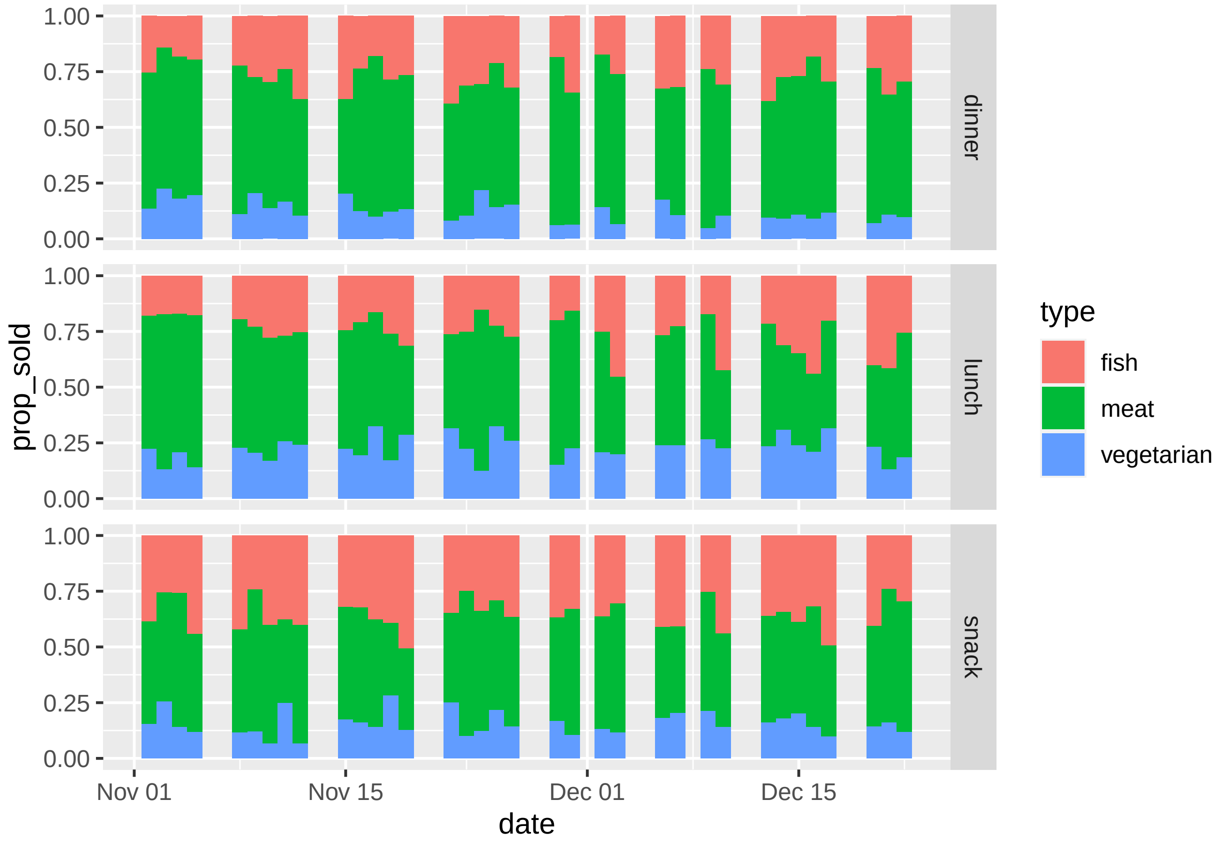

Meals sold by type and time (percentages)

```r
data_intervention %>%
  ggplot(aes(x = date, y = prop_sold, fill = type)) +
  geom_col(width = 1) +
  facet_grid(meal ~ .)
```

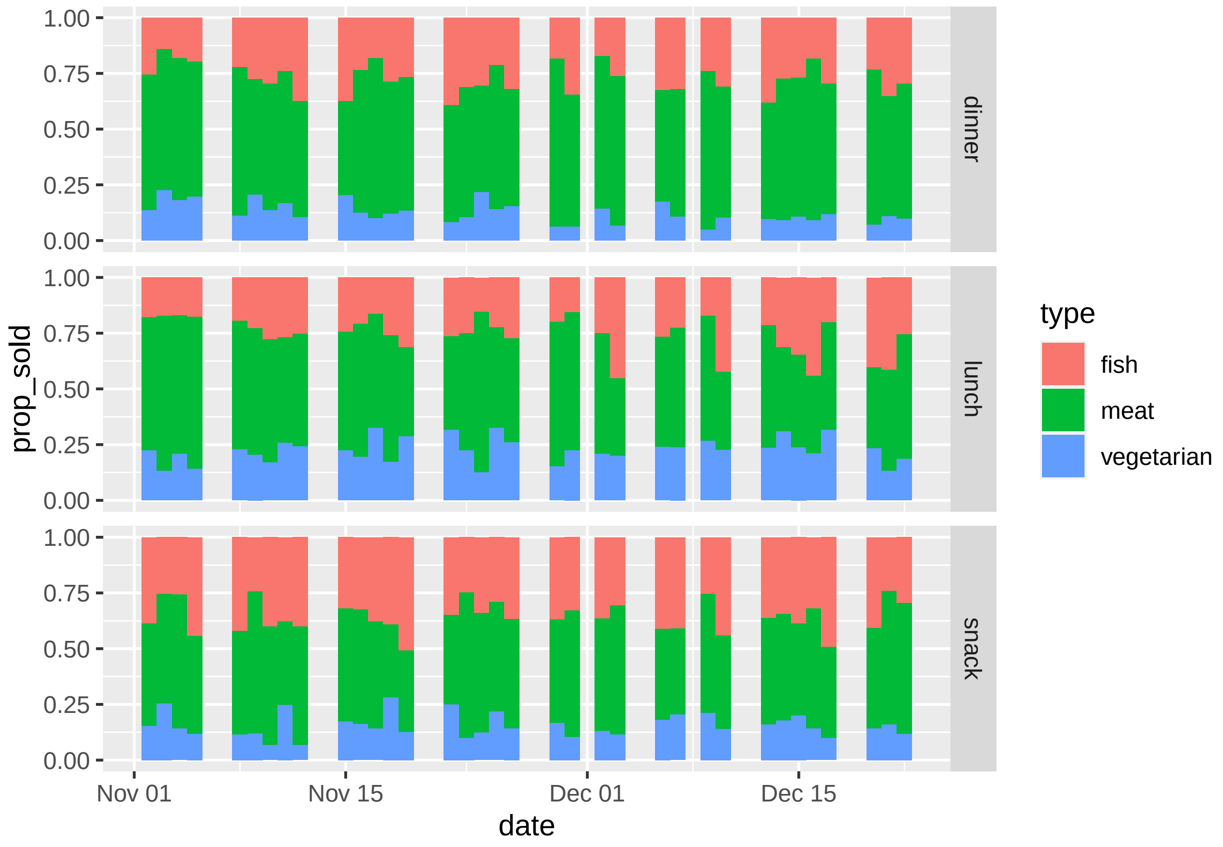

Tables

```r
data_intervention %>%
  group_by(type) %>%
  summarise(
    total = sum(sold, na.rm = TRUE),
    prop = sum(sold, na.rm = TRUE)/sum(total_sold, na.rm = TRUE)
  ) %>% knitr::kable()
```

type	total	prop
fish	4731	0.267
meat	9400	0.530
vegetarian	3604	0.203

```r
data_intervention %>%
  group_by(type, period) %>%
  summarise(
    total = sum(sold, na.rm = TRUE),
```

```
    prop = sum(sold, na.rm = TRUE)/sum(total_sold, na.rm = TRUE)
  ) %>% knitr::kable()
```

type	period	total	prop
fish	pre	1940	0.244
fish	intervention	671	0.255
fish	post	2120	0.297
meat	pre	4456	0.559
meat	intervention	1380	0.524
meat	post	3564	0.500
vegetarian	pre	1569	0.197
vegetarian	intervention	584	0.222
vegetarian	post	1451	0.203

```
data_intervention %>%
  group_by(meal, type) %>%
  summarise(
    total = sum(sold, na.rm = TRUE),
    prop = sum(sold, na.rm = TRUE)/sum(total_sold, na.rm = TRUE)
  ) %>% knitr::kable()
```

meal	type	total	prop
dinner	fish	606	0.267
dinner	meat	1367	0.602
dinner	vegetarian	298	0.131
lunch	fish	3282	0.251
lunch	meat	6855	0.524
lunch	vegetarian	2935	0.225
snack	fish	843	0.352
snack	meat	1178	0.492
snack	vegetarian	371	0.155

Only lunch:

```
data_intervention %>% filter(meal == "lunch") %>%
  group_by(type, period) %>%
  summarise(
    total = sum(sold, na.rm = TRUE),
    prop = sum(sold, na.rm = TRUE)/sum(total_sold, na.rm = TRUE)
  ) %>% knitr::kable()
```

type	period	total	prop
fish	pre	1303	0.222
fish	intervention	425	0.231
fish	post	1554	0.290
meat	pre	3326	0.565
meat	intervention	950	0.517

type	period	total	prop
meat	post	2579	0.482
vegetarian	pre	1253	0.213
vegetarian	intervention	461	0.251
vegetarian	post	1221	0.228

Analysis

Primary, preregistered model

Looking only at the proportion of vegetarian meals at lunch:

```
model_fe <- glm(
  formula = prop_sold ~ period,
  weights = total_sold,
  family = "binomial",
  data = data_intervention %>% filter(type == "vegetarian", meal == "lunch")
)

summary(model_fe)
```

```
##
## Call:
## glm(formula = prop_sold ~ period, family = "binomial", data = data_intervention %>%
##     filter(type == "vegetarian", meal == "lunch"), weights = total_sold)
##
## Deviance Residuals:
##     Min       1Q   Median       3Q      Max
## -5.964   -1.271   0.174   1.006   4.790
##
## Coefficients:
##                      Estimate Std. Error z value Pr(>|z|)
## (Intercept)          -1.3068     0.0318  -41.04  < 2e-16 ***
## periodintervention    0.2140     0.0625    3.42  0.00062 ***
## periodpost            0.0875     0.0456    1.92  0.05484 .
## ---
## Signif. codes:  0 '***' 0.001 '**' 0.01 '*' 0.05 '.' 0.1 ' ' 1
##
## (Dispersion parameter for binomial family taken to be 1)
##
##     Null deviance: 210.18  on 34  degrees of freedom
## Residual deviance: 198.03  on 32  degrees of freedom
##   (2 observations deleted due to missingness)
## AIC: 412.6
##
## Number of Fisher Scoring iterations: 4
```

```
model_fe %>%
  gtsummary::tbl_regression(exponentiate = TRUE, intercept = TRUE) %>%
  gtsummary::modify_column_unhide(column = std.error)
```

Characteristic	OR	SE	95% CI	p-value
(Intercept)	0.27	0.032	0.25, 0.29	<0.001
period				
pre	—	—	—	
intervention	1.24	0.063	1.10, 1.40	<0.001
post	1.09	0.046	1.00, 1.19	0.055

```
emmeans::emmeans(model_fe, specs = ~period, type = "response") %>% knitr::kable()
```

period	prob	SE	df	asymp.LCL	asymp.UCL
pre	0.213	0.005	Inf	0.203	0.224
intervention	0.251	0.010	Inf	0.232	0.271
post	0.228	0.006	Inf	0.217	0.239

Of interest, there was a statistically significant difference in the proportion of vegetarian meals consumed the intervention period as compared to the pre-intervention period (OR = 1.24, 95% CI[1.10, 1.40], p < .001). The change in proportion from pre- to post-intervention was not significant (OR = 1.09, 95% CI[1.00, 1.19], p = .055).

Multilevel models

The following model uses the proportion of vegetarian meals as the dependent variable. All intervention data is used to fit the model (meaning "dinner" and "snack" are included), and allows the intercept to vary with meal type.

```
model_ri <- lme4::glmer(
  formula = prop_sold ~ period + (1 | meal),
  weights = total_sold,
  family = "binomial",
  data = data_intervention %>% filter(type == "vegetarian")
)

summary(model_ri)
```

```
## Generalized linear mixed model fit by maximum likelihood (Laplace
##   Approximation) [glmerMod]
##  Family: binomial  ( logit )
## Formula: prop_sold ~ period + (1 | meal)
##    Data: data_intervention %>% filter(type == "vegetarian")
## Weights: total_sold
##
##      AIC      BIC   logLik deviance df.resid
##      791      802     -392      783      101
##
## Scaled residuals:
##    Min     1Q Median     3Q    Max
## -5.443 -0.887 -0.206  0.663  4.806
##
## Random effects:
```

```
##  Groups Name        Variance Std.Dev.
##  meal   (Intercept) 0.0751   0.274
## Number of obs: 105, groups:  meal, 3
##
## Fixed effects:
##                     Estimate Std. Error z value Pr(>|z|)
## (Intercept)          -1.6453     0.1622  -10.14   <2e-16 ***
## periodintervention    0.1728     0.0550    3.14   0.0017 **
## periodpost            0.0317     0.0409    0.78   0.4381
## ---
## Signif. codes:  0 '***' 0.001 '**' 0.01 '*' 0.05 '.' 0.1 ' ' 1
##
## Correlation of Fixed Effects:
##             (Intr) prdntr
## perdntrvntn -0.094
## periodpost  -0.119  0.355
```

```
model_ri %>%
  gtsummary::tbl_regression(exponentiate = TRUE, tidy_fun = broom.mixed::tidy) %>%
  gtsummary::modify_column_unhide(column = std.error)
```

**Characteristic**	**OR**	**SE**	**95% CI**	**p-value**
period				
pre	—	—	—	
intervention	1.19	0.065	1.07, 1.32	0.002
post	1.03	0.042	0.95, 1.12	0.4
meal.sd___(Intercept)	0.27			

```
emmeans::emmeans(model_ri, specs = ~period, type = "response") %>% knitr::kable()
```

period	prob	SE	df	asymp.LCL	asymp.UCL
pre	0.162	0.022	Inf	0.123	0.210
intervention	0.187	0.025	Inf	0.142	0.241
post	0.166	0.023	Inf	0.127	0.215

Of interest, there was a statistically significant difference in the proportion of vegetarian meals consumed the intervention period as compared to the pre-intervention period (OR = 1.19, 95% CI[1.07, 1.32], p = .002). The change in proportion from pre- to post-intervention was not significant (OR = 1.03, 95% CI[0.95 1.12], p = .4).

The following model also estimates varying slopes by meal type, allowing the effect of period to vary with meal type. However, the model shows a "singular fit" warning, so we don't interpret the results.

```
model_ri_rs <- lme4::glmer(
  formula = prop_sold ~ period + (1 + period | meal),
  weights = total_sold,
  family = "binomial",
  data = data_intervention %>% filter(type == "vegetarian")
)
```

```
## boundary (singular) fit: see help('isSingular')
```

```
summary(model_ri_rs)
```

```
## Generalized linear mixed model fit by maximum likelihood (Laplace
##   Approximation) [glmerMod]
##  Family: binomial  ( logit )
## Formula: prop_sold ~ period + (1 + period | meal)
##    Data: data_intervention %>% filter(type == "vegetarian")
## Weights: total_sold
##
##      AIC      BIC   logLik deviance df.resid
##      790      814     -386      772       96
##
## Scaled residuals:
##    Min     1Q Median     3Q    Max
## -5.489 -0.933 -0.137  0.811  5.104
##
## Random effects:
##  Groups Name               Variance Std.Dev. Corr
##  meal   (Intercept)        0.03948  0.1987
##         periodintervention 0.00946  0.0973   1.00
##         periodpost         0.03141  0.1772   1.00 1.00
## Number of obs: 105, groups:  meal, 3
##
## Fixed effects:
##                    Estimate Std. Error z value Pr(>|z|)
## (Intercept)         -1.5711     0.1221  -12.87   <2e-16 ***
## periodintervention   0.0864     0.0962    0.90     0.37
## periodpost          -0.1345     0.1217   -1.11     0.27
## ---
## Signif. codes:  0 '***' 0.001 '**' 0.01 '*' 0.05 '.' 0.1 ' ' 1
##
## Correlation of Fixed Effects:
##             (Intr) prdntr
## perdntrvntn 0.404
## periodpost  0.671  0.635
## optimizer (Nelder_Mead) convergence code: 0 (OK)
## boundary (singular) fit: see help('isSingular')
```

Sensitivity analyses

We report the primary model, run three times:

1. Excluding the first Monday of intervention (November 22)
2. Excluding the last three days (of data) in December (20-22)
3. Excluding all days mentioned in points 1 and 2.

```
data_sensitivity_1 <- data_intervention %>%
  filter(date != "2021-11-22")

data_sensitivity_2 <- data_intervention %>%
```

```
  filter(date < "2021-12-20")

data_sensitivity_3 <- data_intervention %>%
  filter(date != "2021-11-22", date < "2021-12-20")

model_fe <- glm(
  formula = prop_sold ~ period,
  weights = total_sold,
  family = "binomial",
  data = data_sensitivity_1 %>% filter(type == "vegetarian", meal == "lunch")
)

model_fe %>%
  gtsummary::tbl_regression(exponentiate = TRUE, intercept = TRUE) %>%
  gtsummary::modify_column_unhide(column = std.error)
```

**Characteristic**	**OR**	**SE**	**95% CI**	**p-value**
(Intercept)	0.27	0.032	0.25, 0.29	<0.001
period				
pre	—	—	—	
intervention	1.14	0.069	1.00, 1.31	0.050
post	1.09	0.046	1.00, 1.19	0.055

```
emmeans::emmeans(model_fe, specs = ~period, type = "response") %>% knitr::kable()
```

period	prob	SE	df	asymp.LCL	asymp.UCL
pre	0.213	0.005	Inf	0.203	0.224
intervention	0.236	0.011	Inf	0.216	0.259
post	0.228	0.006	Inf	0.217	0.239

```
model_fe <- glm(
  formula = prop_sold ~ period,
  weights = total_sold,
  family = "binomial",
  data = data_sensitivity_2 %>% filter(type == "vegetarian", meal == "lunch")
)

model_fe %>% gtsummary::tbl_regression(exponentiate = TRUE, intercept = TRUE) %>%
  gtsummary::modify_column_unhide(column = std.error)
```

**Characteristic**	**OR**	**SE**	**95% CI**	**p-value**
(Intercept)	0.27	0.032	0.25, 0.29	<0.001
period				
pre	—	—	—	
intervention	1.24	0.063	1.10, 1.40	<0.001
post	1.13	0.047	1.03, 1.24	0.011

```
emmeans::emmeans(model_fe, specs = ~period, type = "response") %>% knitr::kable()
```

period	prob	SE	df	asymp.LCL	asymp.UCL
pre	0.213	0.005	Inf	0.203	0.224
intervention	0.251	0.010	Inf	0.232	0.271
post	0.234	0.006	Inf	0.222	0.246

```
model_fe <- glm(
  formula = prop_sold ~ period,
  weights = total_sold,
  family = "binomial",
  data = data_sensitivity_3 %>% filter(type == "vegetarian", meal == "lunch")
)

model_fe %>% gtsummary::tbl_regression(exponentiate = TRUE, intercept = TRUE) %>%
  gtsummary::modify_column_unhide(column = std.error)
```

**Characteristic**	**OR**	**SE**	**95% CI**	**p-value**
(Intercept)	0.27	0.032	0.25, 0.29	<0.001
period				
pre	—	—	—	
intervention	1.14	0.069	1.00, 1.31	0.050
post	1.13	0.047	1.03, 1.24	0.011

```
emmeans::emmeans(model_fe, specs = ~period, type = "response") %>% knitr::kable()
```

period	prob	SE	df	asymp.LCL	asymp.UCL
pre	0.213	0.005	Inf	0.203	0.224
intervention	0.236	0.011	Inf	0.216	0.259
post	0.234	0.006	Inf	0.222	0.246

In summary, the relevant contrast remains significant when we exclude the last three days of December (2) but not when we (also) exclude the first Monday of intervention (1, 3).

Now the secondary (multilevel) model, also run three times:

```
model_ri <- lme4::glmer(
  formula = prop_sold ~ period + (1 | meal),
  weights = total_sold,
  family = "binomial",
  data = data_sensitivity_1 %>% filter(type == "vegetarian")
)

model_ri %>% gtsummary::tbl_regression(exponentiate = TRUE, tidy_fun = broom.mixed::tidy) %>%
  gtsummary::modify_column_unhide(column = std.error)
```

Characteristic	OR	SE	95% CI	p-value
period				
pre	—	—	—	
intervention	1.11	0.067	0.99, 1.25	0.075
post	1.03	0.042	0.95, 1.12	0.4
meal.sd___(Intercept)	0.26			

```
emmeans::emmeans(model_ri, specs = ~period, type = "response") %>% knitr::kable()
```

period	prob	SE	df	asymp.LCL	asymp.UCL
pre	0.163	0.021	Inf	0.125	0.209
intervention	0.178	0.024	Inf	0.136	0.229
post	0.167	0.022	Inf	0.129	0.214

```
model_ri <- lme4::glmer(
  formula = prop_sold ~ period + (1 | meal),
  weights = total_sold,
  family = "binomial",
  data = data_sensitivity_2 %>% filter(type == "vegetarian")
)

model_ri %>% gtsummary::tbl_regression(exponentiate = TRUE, tidy_fun = broom.mixed::tidy) %>%
  gtsummary::modify_column_unhide(column = std.error)
```

Characteristic	OR	SE	95% CI	p-value
period				
pre	—	—	—	
intervention	1.19	0.065	1.07, 1.32	0.002
post	1.06	0.045	0.98, 1.16	0.14
meal.sd___(Intercept)	0.27			

```
emmeans::emmeans(model_ri, specs = ~period, type = "response") %>% knitr::kable()
```

period	prob	SE	df	asymp.LCL	asymp.UCL
pre	0.162	0.022	Inf	0.124	0.209
intervention	0.187	0.025	Inf	0.143	0.241
post	0.171	0.023	Inf	0.130	0.220

```
model_ri <- lme4::glmer(
  formula = prop_sold ~ period + (1 | meal),
  weights = total_sold,
  family = "binomial",
  data = data_sensitivity_3 %>% filter(type == "vegetarian")
)

model_ri %>% gtsummary::tbl_regression(exponentiate = TRUE, tidy_fun = broom.mixed::tidy) %>%
  gtsummary::modify_column_unhide(column = std.error)
```

**Characteristic**	**OR**	**SE**	**95% CI**	**p-value**
period				
pre	—	—	—	
intervention	1.11	0.067	0.99, 1.25	0.076
post	1.06	0.045	0.98, 1.16	0.14
meal.sd___(Intercept)	0.26			

```
emmeans::emmeans(model_ri, specs = ~period, type = "response") %>% knitr::kable()
```

period	prob	SE	df	asymp.LCL	asymp.UCL
pre	0.163	0.021	Inf	0.126	0.209
intervention	0.178	0.024	Inf	0.136	0.229
post	0.172	0.022	Inf	0.132	0.219

Again, the relevant contrast remains significant when we exclude the last three days of December (2) but not when we (also) exclude the first Monday of intervention (1, 3).

Session info

```
sessioninfo::session_info()
```

```
## - Session info ---------------------------------------------------------------
##  setting  value
##  version  R version 4.2.1 (2022-06-23 ucrt)
##  os       Windows 10 x64 (build 19044)
##  system   x86_64, mingw32
##  ui       RTerm
##  language (EN)
##  collate  English_United States.utf8
##  ctype    English_United States.utf8
##  tz       Europe/London
##  date     2022-10-13
##  pandoc   2.18 @ C:/Program Files/RStudio/bin/quarto/bin/tools/ (via rmarkdown)
##
## - Packages -------------------------------------------------------------------
##  package       * version date (UTC) lib source
##  assertthat      0.2.1   2019-03-21 [1] CRAN (R 4.2.1)
##  backports       1.4.1   2021-12-13 [1] CRAN (R 4.2.0)
##  boot            1.3-28  2021-05-03 [2] CRAN (R 4.2.1)
##  broom           1.0.0   2022-07-01 [1] CRAN (R 4.2.1)
##  broom.helpers   1.8.0   2022-07-05 [1] CRAN (R 4.2.1)
##  broom.mixed   * 0.2.9.4 2022-04-17 [1] CRAN (R 4.2.1)
##  cellranger      1.1.0   2016-07-27 [1] CRAN (R 4.2.1)
##  cli             3.3.0   2022-04-25 [1] CRAN (R 4.2.1)
##  coda            0.19-4  2020-09-30 [1] CRAN (R 4.2.1)
##  codetools       0.2-18  2020-11-04 [2] CRAN (R 4.2.1)
##  colorspace      2.0-3   2022-02-21 [1] CRAN (R 4.2.1)
##  crayon          1.5.1   2022-03-26 [1] CRAN (R 4.2.1)
```

```
##  DBI             1.1.3   2022-06-18 [1] CRAN (R 4.2.1)
##  dbplyr          2.2.1   2022-06-27 [1] CRAN (R 4.2.1)
##  digest          0.6.29  2021-12-01 [1] CRAN (R 4.2.1)
##  dplyr         * 1.0.9   2022-04-28 [1] CRAN (R 4.2.1)
##  ellipsis        0.3.2   2021-04-29 [1] CRAN (R 4.2.1)
##  emmeans       * 1.7.5   2022-06-22 [1] CRAN (R 4.2.1)
##  estimability    1.4     2022-07-03 [1] CRAN (R 4.2.1)
##  evaluate        0.15    2022-02-18 [1] CRAN (R 4.2.1)
##  fansi           1.0.3   2022-03-24 [1] CRAN (R 4.2.1)
##  farver          2.1.1   2022-07-06 [1] CRAN (R 4.2.1)
##  fastmap         1.1.0   2021-01-25 [1] CRAN (R 4.2.1)
##  forcats       * 0.5.1   2021-01-27 [1] CRAN (R 4.2.1)
##  fs              1.5.2   2021-12-08 [1] CRAN (R 4.2.1)
##  furrr           0.3.1   2022-08-15 [1] CRAN (R 4.2.1)
##  future          1.27.0  2022-07-22 [1] CRAN (R 4.2.1)
##  gargle          1.2.0   2021-07-02 [1] CRAN (R 4.2.1)
##  generics        0.1.3   2022-07-05 [1] CRAN (R 4.2.1)
##  ggplot2       * 3.3.6   2022-05-03 [1] CRAN (R 4.2.1)
##  globals         0.15.1  2022-06-24 [1] CRAN (R 4.2.1)
##  glue            1.6.2   2022-02-24 [1] CRAN (R 4.2.1)
##  googledrive     2.0.0   2021-07-08 [1] CRAN (R 4.2.1)
##  googlesheets4   1.0.0   2021-07-21 [1] CRAN (R 4.2.1)
##  gt              0.6.0   2022-05-24 [1] CRAN (R 4.2.1)
##  gtable          0.3.0   2019-03-25 [1] CRAN (R 4.2.1)
##  gtsummary     * 1.6.1   2022-06-22 [1] CRAN (R 4.2.1)
##  haven           2.5.0   2022-04-15 [1] CRAN (R 4.2.1)
##  here          * 1.0.1   2020-12-13 [1] CRAN (R 4.2.1)
##  highr           0.9     2021-04-16 [1] CRAN (R 4.2.1)
##  hms             1.1.1   2021-09-26 [1] CRAN (R 4.2.1)
##  htmltools       0.5.3   2022-07-18 [1] CRAN (R 4.2.1)
##  httr            1.4.3   2022-05-04 [1] CRAN (R 4.2.1)
##  jsonlite        1.8.0   2022-02-22 [1] CRAN (R 4.2.1)
##  knitr         * 1.39    2022-04-26 [1] CRAN (R 4.2.1)
##  labeling        0.4.2   2020-10-20 [1] CRAN (R 4.2.0)
##  labelled        2.9.1   2022-05-05 [1] CRAN (R 4.2.1)
##  lattice         0.20-45 2021-09-22 [2] CRAN (R 4.2.1)
##  lifecycle       1.0.1   2021-09-24 [1] CRAN (R 4.2.1)
##  listenv         0.8.0   2019-12-05 [1] CRAN (R 4.2.1)
##  lme4          * 1.1-30  2022-07-08 [1] CRAN (R 4.2.1)
##  lubridate       1.8.0   2021-10-07 [1] CRAN (R 4.2.1)
##  magrittr        2.0.3   2022-03-30 [1] CRAN (R 4.2.1)
##  MASS            7.3-58  2022-07-14 [1] CRAN (R 4.2.1)
##  Matrix        * 1.5-1   2022-09-13 [1] CRAN (R 4.2.1)
##  minqa           1.2.4   2014-10-09 [1] CRAN (R 4.2.1)
##  modelr          0.1.8   2020-05-19 [1] CRAN (R 4.2.1)
##  multcomp        1.4-19  2022-04-26 [1] CRAN (R 4.2.1)
##  munsell         0.5.0   2018-06-12 [1] CRAN (R 4.2.1)
##  mvtnorm         1.1-3   2021-10-08 [1] CRAN (R 4.2.0)
##  nlme            3.1-157 2022-03-25 [2] CRAN (R 4.2.1)
##  nloptr          2.0.3   2022-05-26 [1] CRAN (R 4.2.1)
##  parallelly      1.32.1  2022-07-21 [1] CRAN (R 4.2.1)
##  pillar          1.8.0   2022-07-18 [1] CRAN (R 4.2.1)
##  pkgconfig       2.0.3   2019-09-22 [1] CRAN (R 4.2.1)
##  purrr         * 0.3.4   2020-04-17 [1] CRAN (R 4.2.1)
```

```
##  R6              2.5.1   2021-08-19 [1] CRAN (R 4.2.1)
##  Rcpp            1.0.9   2022-07-08 [1] CRAN (R 4.2.1)
##  readr         * 2.1.2   2022-01-30 [1] CRAN (R 4.2.1)
##  readxl          1.4.0   2022-03-28 [1] CRAN (R 4.2.1)
##  reprex          2.0.1   2021-08-05 [1] CRAN (R 4.2.1)
##  rlang           1.0.4   2022-07-12 [1] CRAN (R 4.2.1)
##  rmarkdown       2.14    2022-04-25 [1] CRAN (R 4.2.1)
##  rprojroot       2.0.3   2022-04-02 [1] CRAN (R 4.2.1)
##  rstudioapi      0.13    2020-11-12 [1] CRAN (R 4.2.1)
##  rvest           1.0.2   2021-10-16 [1] CRAN (R 4.2.1)
##  sandwich        3.0-2   2022-06-15 [1] CRAN (R 4.2.1)
##  scales          1.2.0   2022-04-13 [1] CRAN (R 4.2.1)
##  sessioninfo   * 1.2.2   2021-12-06 [1] CRAN (R 4.2.1)
##  stringi         1.7.8   2022-07-11 [1] CRAN (R 4.2.1)
##  stringr       * 1.4.0   2019-02-10 [1] CRAN (R 4.2.1)
##  survival        3.3-1   2022-03-03 [2] CRAN (R 4.2.1)
##  TH.data         1.1-1   2022-04-26 [1] CRAN (R 4.2.1)
##  tibble        * 3.1.8   2022-07-22 [1] CRAN (R 4.2.1)
##  tidyr         * 1.2.0   2022-02-01 [1] CRAN (R 4.2.1)
##  tidyselect      1.1.2   2022-02-21 [1] CRAN (R 4.2.1)
##  tidyverse     * 1.3.2   2022-07-18 [1] CRAN (R 4.2.1)
##  tzdb            0.3.0   2022-03-28 [1] CRAN (R 4.2.1)
##  utf8            1.2.2   2021-07-24 [1] CRAN (R 4.2.1)
##  vctrs           0.4.1   2022-04-13 [1] CRAN (R 4.2.1)
##  withr           2.5.0   2022-03-03 [1] CRAN (R 4.2.1)
##  xfun            0.31    2022-05-10 [1] CRAN (R 4.2.1)
##  xml2            1.3.3   2021-11-30 [1] CRAN (R 4.2.1)
##  xtable          1.8-4   2019-04-21 [1] CRAN (R 4.2.1)
##  yaml            2.3.5   2022-02-21 [1] CRAN (R 4.2.0)
##  zoo             1.8-10  2022-04-15 [1] CRAN (R 4.2.1)
##
##  [1] C:/Users/admin/AppData/Local/R/win-library/4.2
##  [2] C:/Program Files/R/R-4.2.1/library
##
## ------------------------------------------------------------------------------
```
